# Vascular-Derived Vegfa Promotes Cortical Interneuron Migration and Proximity to the Vasculature in the Developing Forebrain

**DOI:** 10.1093/cercor/bhy082

**Published:** 2018-04-18

**Authors:** Melissa Barber, William D Andrews, Fani Memi, Phillip Gardener, Daniel Ciantar, Mathew Tata, Christiana Ruhrberg, John G Parnavelas

**Affiliations:** 1 Department of Cell and Developmental Biology, University College London, London, UK; 2 Institute of Ophthalmology, University College London, London, UK

**Keywords:** cortical development, GABAergic interneurons, neurovascular coupling, *Vegfa*

## Abstract

Vascular endothelial growth factor (*Vegfa*) is essential for promoting the vascularization of the embryonic murine forebrain. In addition, it directly influences neural development, although its role in the forming forebrain is less well elucidated. It was recently suggested that *Vegfa* may influence the development of GABAergic interneurons, inhibitory cells with crucial signaling roles in cortical neuronal circuits. However, the mechanism by which it affects interneuron development remains unknown. Here we investigated the developmental processes by which *Vegfa* may influence cortical interneuron development by analyzing transgenic mice that ubiquitously express the Vegfa120 isoform to perturb its signaling gradient. We found that interneurons reach the dorsal cortex at mid phases of corticogenesis despite an aberrant vascular network. Instead, endothelial ablation of Vegfa alters cortical interneuron numbers, their intracortical distribution and spatial proximity to blood vessels. We show for the first time that vascular-secreted guidance factors promote early-migrating interneurons in the intact forebrain in vivo and identify a novel role for vascular-Vegfa in this process.

## Introduction

The mammalian embryonic forebrain develops encased by a dense vascular perineural plexus (PNP), which gives rise to a molecularly and anatomically distinct periventricular plexus (PVP) that invades the basal ganglia primordium at the onset of neurogenesis (Vasudevan et al. 2008; Marin-Padilla and Knopman 2011). The PVP subsequently grows along a ventral-dorsal gradient to flank the germinal domains of the dorsal cortex and mirrors the neurogenic gradient (Vasudevan et al. 2008; Javaherian and Kriegstein 2009). The intimate association of blood vessels with the nervous system is essential to meet the homeostatic demands of the developing brain, but there is increasing evidence that the vasculature can directly affect neurogenesis and cell migration (Tan et al. 2016; Tsai et al. 2016). The PVP, for example, was recently shown to regulate the generation of GABAergic interneurons, inhibitory cells with crucial functions in modulating cortical circuitry (Tan et al. 2016). Interneurons are generated predominantly in the medial ganglionic eminence (MGE) in the ventral forebrain (Lavdas et al. 1999; Sussel et al. 1999; Wonders et al. 2008) and migrate over long distances to reach the forming cortical plate (CP), where they assemble with their pyramidal cell counterparts into cortical circuits. Deficits in the number, migration and positioning of cortical interneurons are suggested to result in an imbalance of excitatory and inhibitory activity associated with neurological and psychiatric disorders such as epilepsy, autism and schizophrenia (Rubenstein and Merzenich 2003; Murray et al. 2014; Lazarus et al. 2015; Inan et al. 2016). Thus, understanding how the vasculature influences interneuron development is crucial for furthering our knowledge of the pleiotropic origins of neurodevelopmental disorders, especially those that result from an imbalance of excitatory and inhibitory cortical neural signaling.

A key molecule coordinating vascular and neural development is the pro-angiogenic factor *Vegfa*. *Vegfa* acts on blood vessels to promote their ingression and growth within the forebrain parenchyma through paracrine signaling from neural progenitors (Haigh et al. 2003; Raab et al. 2004). This growth factor is post-transcriptionally spliced into 3 prevalent isoforms which differ in their matrix-binding affinities, as determined by the presence (Vegfa165, Vegfa188) or absence (Vegfa120) of a heparan sulfate proteoglycan binding domain. Together, Vegfa isoforms form an extracellular gradient crucial for the correct outgrowth and branching of blood vessels (Ruhrberg et al. 2002). A vascular source of Vegfa also maintains the homeostasis and survival of blood vessels postnatally through autocrine signaling (Lee et al. 2007; Domigan et al. 2015). In addition to its role in vascular development, Vegfa directly influences neurogenesis in the adult hippocampus (Fournier et al. 2012), neuronal migration in the cerebellum and hindbrain (Schwarz et al. 2004; Ruiz de Almodovar et al. 2010; Tillo et al. 2015), and exerts a pro-survival role on migrating neuroendocrine cells (Cariboni et al. 2011). While a pleiotropic role for Vegfa in neural development is well established, it is not known whether it influences cortical interneuron development directly or indirectly through its action on the vasculature. Interestingly, the expression of *distal-less 1 and 2 (Dlx1&2*), essential homeodomain transcription factors required for the correct specification and migration of GABAergic interneurons, was reported to be downregulated in *Vegfa*^*188/188*^ and *Vegfa*^*188/120*^ knock-in mutants, suggesting that Vegfa isoforms may be involved in the generation and specification of interneurons (Darland et al. 2011; Cain et al. 2014). More recently, endothelial cell-ablation of VEGF was suggested to affect corticogenesis and to alter interneuron numbers (Li et al. 2013), however the underlying mechanism was not addressed. Understanding the effects of Vegfa signaling on cortical interneurons is important, as it is expressed in neural and vascular cells in the fetal human brain (Virgintino et al. 2003), and because *Vegfa* polymorphisms and its downregulation in the prefrontal cortex have been implicated in schizophrenia (Fulzele and Pillai 2009; Gao et al. 2015), a neurodevelopmental disorder also associated with interneuron deficits (Murray et al. 2014; Inan et al. 2016).

Here, we analyzed cortical interneuron migration in mice that selectively expressed only the Vegfa120 isoform to perturb the organization of the vascular network while circumventing early-embryonic lethality which occurs upon ubiquitous or neural depletion of all *Vegfa* isoforms (Carmeliet et al. 1999; Haigh et al. 2003), and found that this did not impede interneuron migration at mid phases of cortical development. Instead, depletion of endothelial-*Vegfa* decreased cortical interneuron numbers and altered their intracortical distribution and spatial proximity to blood vessels. Together, this work identifies a novel role for vascular-Vegfa and its isoforms in modulating cortical interneuron numbers and positioning and in promoting their proximity to the vasculature during early phases of their migration.

## Materials and Methods

### Mouse Strains

All experimental procedures were performed in accordance with the UK Animals (Scientific Procedures) Act 1986 and institutional guidelines. Time-mated Sprague Dawley albino rats were provided by UCL Biological Services. Transgenic mouse lines used in this study included *GAD67-GFP* (Δ*neo*) (Tamamaki et al. 2003), *Tie2-GFP*^*+*^(287Sato/J) (Motoike et al. 2000) *Vegf*^*120/120*^ & *Vegf*^*165/165*^ (Carmeliet et al. 1999), and *Tie2Cre;Vegfa*^*fl/fl*^ mice (Gerber et al. 1999; Kisanuki et al. 2001), which were all maintained on a C57/bl6J background. The day the vaginal plug was found was considered as embryonic day (E) 0.5. Animals of both sexes were used in our experiments.

### Immunohistochemistry

Immunohistochemistry was carried out as described previously (Andrews et al. 2008). Dissected embryonic brains were fixed in 4% PFA, cryoprotected overnight in 30% sucrose and frozen embedded in OCT (Tissue Tek). Brains were sectioned on a Cryostat (Bright Instruments) at 25 μm and incubated overnight in one of the following antibodies: rabbit polyclonal anti-calbindin (CB-28; 1:3000; Swant), mouse monoclonal anti-Cd140b/Pdgfrß (1:350, ThermoFisher Scientific), chicken polyclonal raised against GFP (1:500, Aves Labs), rabbit anti-phosphohistone H-3 (PH3; 1:1000, Milipore), anti-cleaved caspase-3 (CC3; 1:250, Cell Signaling Technology), anti-VEGFR1, anti-VEGFR2, anti-VEGFR3 (all 1:500, R&D Systems), rabbit anti-human VEGF (1:350, Abcam), anti-rab Tbr2, anti-mouse Satb2, anti-rat Satb2 (all 1:350, Abcam), and anti-mG10 (1:3000; kind gift from A. Goffinet). For blood vessel staining, sections were incubated with biotinylated *Griffonia (Bandeiraea) Simplicifolia* lectin I (Isolectin B4) (1:200, Vector) followed by fluorescent Strepatividin-405 (1:200, Vector Lab).

### In Situ Hybridization

In situ hybridization was performed as described previously (Andrews et al. 2016). RNA probes *Vegfa-165* (HindIII), *Sdf1/Cxcl12* (SalI), *Gad67* (XhoI), *Nrp1* (NotI), and *Lhx6* (NotI) were generated by linearization of plasmids (with appropriate restriction enzymes), and RNA produced using T7 (*Lhx6, Vegfa165)* or T3 (*Gad67, Lhx6, Sdf1/Cxcl12*) polymerase to obtain antisense probes. Following hybridization, sections incubated overnight in alkaline phosphate conjugated anti-Dig antibody (Roche), followed by Fast Red (Enzo LifeSciences) or BCIP/NBT substrate (Roche) for fluorescent or colorimetric detection.

### qPCR Analysis of FACS-Isolated Cells

qPCR was performed as previously described (Hernandez-Miranda et al. 2011). Total RNA was extracted from FACS-purified: GAD67-GFP^+^ cells taken from the dissociated cortex and MGE at E13.5 and E15.5; Tie2-Gfp^+^ cells were isolated from the dissociated meninges, subpallium and cortex at E14.5 and E18.5 using the QIAGEN RNeasy Plus kit. RNA was treated with DNaseI and cDNA was generated from 25 ng of RNA using the QIAGEN Whole Transcriptome Amplification kit as described in the manufacturer’s protocol. Primers for PCR were designed by Merck-Genosys and were as follows: *Gapdh* (forward, ATGACATCAAGAAGGTGGTG; reverse, CATACCAGGAAATGAGCTTG); *Vegfa120*, *Vegfa165* and *Vegfa188* primer sequences were described previously (Tillo et al. 2015). PCR was performed using Sybr Green reagent (Merck) on a CFX96 Real-Time PCR Detector System (Bio-Rad) and performed in triplicate, with glyceraldehyde-3-phosphate dehydrogenase (GAPDH) used as endogenous reference gene control. Relative quantification was determined by the ΔΔc(t) method (Livak and Schmittgen 2001) or using the BioRad CFX Manager 3.1. FACS analysis was performed at the Rayne Institute flow cytometry facility (UCL) with a BDFACS Aria II sorter (P9C500001), with Gad67-Gfp+ and Tie-Gfp+ cells isolated using the 488-nm excitation laser and a 530/30 band pass filter. Dissociated cells taken from non-Gfp+ embryos were used as a control for fluorescence. TO-PRO^®^-3 Iodide (T3605, Thermofisher) was added to live cell suspensions immediately prior to FAC-sorting, to enable detection of dead cells using the far-red 642nm laser, and to ensure that only viable cells were collected.

### Boyden Chemotaxis Assay

Chemotaxis assays were performed using a 48-well Boyden’s chamber (NeuroProbe) as described previously (Hernandez-Miranda et al. 2011). Dissociated rat or murine MGE cells were suspended in serum-free Optimem medium (ThermoFisher Scientific) (2 × 10^6^ cells/mL) and placed in the open-bottom wells of the upper compartment of open the chamber. For *Vegfa* receptor blocking experiments, Axitinib (1.2 nM, Tocris Bioscience), Vatalanib (50 nM, ApexBio) and Nrp1 blocking antibody (30 μg/mL, R&D Systems) were added to the cell suspension and incubated for 15 min at 37 °C before use. MGE cells were separated from the lower chamber by a polycarbonate porous membrane (8 μm pores) and precoated with laminin (10 μg/mL) and poly-L-lysine (10 μg/ml). 27 μL of chemotactic agents: *Vegfa*120, 50 nM (R&D Systems); *Vegfa*165, 50 nM (R&D Systems); *Vegfa*188, 50 nM (R&D Systems) or control low-serum Optimem media (ThermoFisher Scientific), were placed into the lower compartment of the chamber. For haptotactic Boyden assays, the polycarbonate porous membrane was precoated with *Vegfa*_188_ proteins (50 nM) together with laminin (10 μg/mL) and poly-L-lysine (10 μg/ml) overnight at 4 °C, and 27 μL of Optimem low-serum media added to the bottom wells. The chamber was kept in an incubator at 37°C overnight. Following incubation, the migrated cells that adhered to the underside of the membrane were fixed in ice-cold methanol (VWR) and nuclei stained using the Giemsa stain (ThermoFisher Scientific). Quantifications were carried out using a light microscope, with a 20× or 40× objective, and 3 random fields of stained cells counted for each well, with the mean number of migrating cells per mm^2^ estimated for each experimental condition. All assays were repeated in 3 independent experiments.

### Fiji Plugins to Model Interneuron and Vascular Distribution

The plugins distributed 2 types of object in a 3D space (i.e., an image stack), with dots used to represent interneurons, and lines or pairs of parallel lines used to represent blood vessels. The 2 object types are distributed randomly and the plugin calculated the distance from the center of each dot to the nearest point on a line to represent the minimum interneuron-blood vessel distance in arbitrary units. To simulate area fraction occupancy of blood vessels, a modification of the plugin restricts the line-pairs (i.e., “vessels”) to a user-specified fraction of the 3D space. For chemo-attraction models simulating migration, the dots & single lines are distributed randomly, with dots moving up “chemoattractant gradients” to simulate migration. The gradients are created from each line and decrease in “intensity” based on one of the following:

i/. LINEAR:Cn=C0−(C0∗n∗Dr)

Where:

Cn-Amount of “chemoattractant” at pixel n.

C0-Amount of “chemoattractant” at pixel 0 (i.e., pixels touching the line).

*n*-Distance from line.

Dr-Decay rate.

ii/. CONSTANTDECREMENTCn=Cn−1∗Dr

Where:

Cn-1-Amount of “chemoattractant” at pixel (*n*−1).

iii/. EXPONENTIAL:Cn=C0∗exp(−Dr∗n)

iv/. SIGMOID:Cn=C0/(1+exp(−Dr∗(HL−n)))

Where:

HL-Half-length (the mid-point of the sloping part of the sigmoid curve).

Each model was run 100 times (or 20 times for the “migration” model) for statistical analysis of intra-object distances as assessed using the KS-test.

### Analyses of Vascular Surface Area, Cell Counts, and Colocalization Studies Using Imaris Software

Vascular surface area was measured by generating reconstructed vascular surfaces using the Imaris surface rendering module, in which blood vessels were thresholded according to signal intensity and predicted cell size and total surface area measured. Cell counts and intercellular distances were analyzed by thresholding cells according to signal intensity of cell markers and predicted cell size using the spot module. Colocalization analysis was carried out by thresholding signal intensity in 2 channels and plotting pairs of voxel intensities in a histogram. Colocalization masks were generated using the Imaris colocalization module which corresponded to voxels in which signal from both channels colocalised, and images false-colored in magenta.

### Statistics

Statistical analyses were performed by Microsoft Excel software. All data are reported as mean number ± SEM. The statistical significance between pairs of group means was tested by Student’s *t*-test, with a one-way analysis of variance used to compare multiple group means using GraphPad Prism software. Data generated from the bespoke plugin to measure differences between spatial proximity of interneurons relative to blood vessels were plotted for different groups, with cumulative distribution of measured minimum distances obtained plotted for different groups, and the differences between their distributions assessed with the Kolmologrov–Smirnov statistical test. Significance was set at a value of *P* ≤ 0.05.

## Results

### MGE Progenitors and Migrating Interneurons Are Located near Multiple Cellular Sources of Vegfa and Express Cognate Receptors

**Figure 1.**
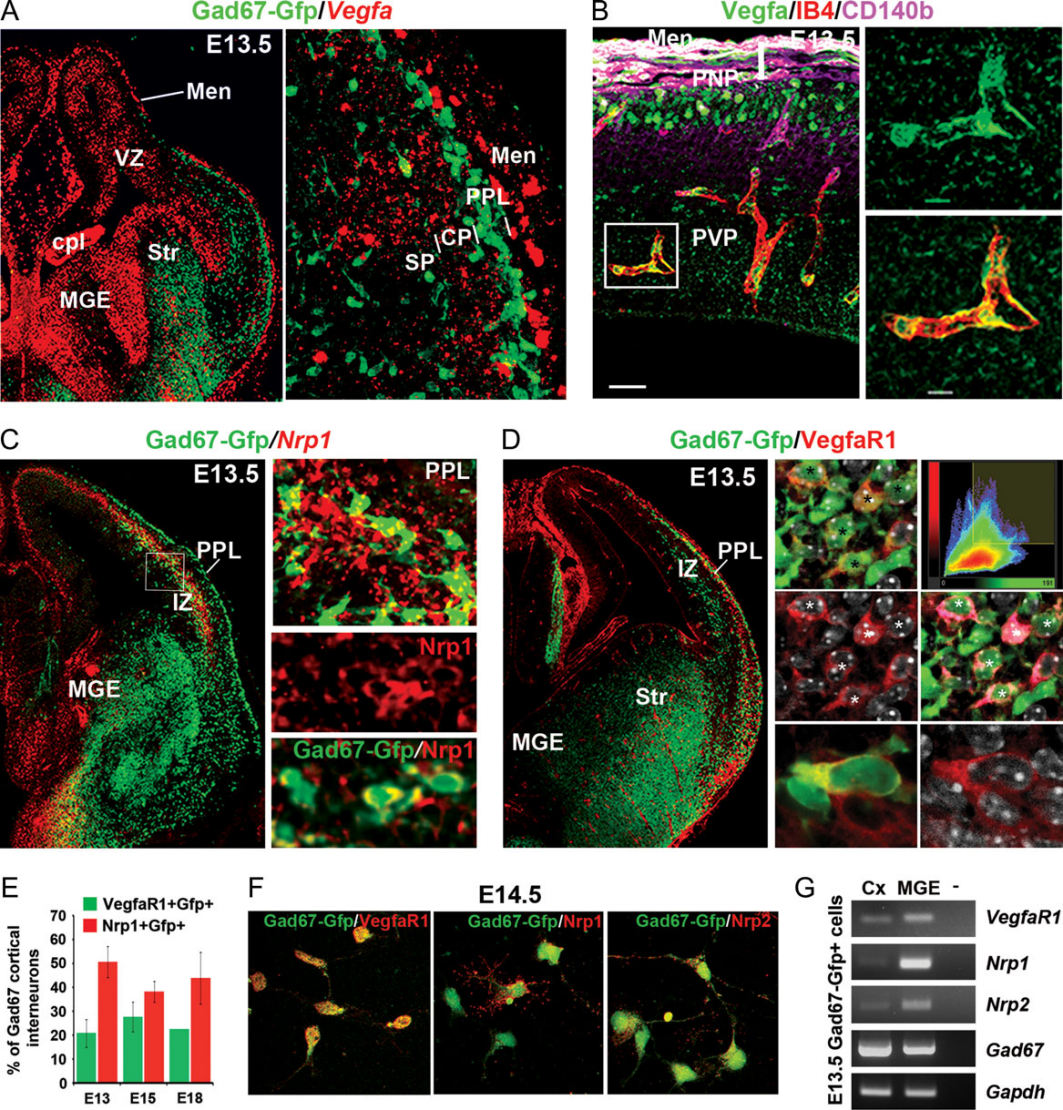
MGE progenitors and migrating cortical interneurons are located close to multiple cellular sources of Vegfa in the developing forebrain and express cognate receptors. (*A*) *Vegfa mRNA* expression in the MGE and in the dorsal VZ and meninges adjacent to deep and superficial Gad67-Gfp^+^ interneuron migratory streams in the E13.5 Gad67-Gfp^+^ mouse cortex. Insert shows high magnification of the cortex with strong localization of *Vegfa* in the meninges (Men) and at lower levels of expression in the forming CP. (*B*) Immunolocalization of Vegfa protein, the vascular endothelial marker IB4, and the pericyte-specific Pdgfrß/Cd140b protein in the E13.5 dorsal-lateral cortex. (*C*) In situ hybridization for *Nrp1* in the E13.5 Gad67-Gfp^+^ mouse forebrain with colocalization of Nrp1 protein in Gad67-Gfp+ interneurons shown in bottom 2 inserts. (*D*) Imunostaining for VegfaR1 in the E13.5 Gad67-Gfp^+^ mouse forebrain with right panels comprising single optical confocal slices showing colocalization of VegfaR1 in Gad67-Gfp^+^ cells (denoted by stars). Histogram plots the distribution of pairs of voxel intensities for Gad67-Gfp (green channel) and VegfaR1 (red channel) (top right panel), with colocalised signal corresponding to false-colored magenta (right middle panel). Bottom panel shows high magnification of VegfaR1 (red) distributed around the cell soma of a Gad67-gfp^+^ interneuron (green) where these colocalise (yellow). (*E*) Quantifications of the percentage of Gad67-Gfp^+^ cortical interneurons which express VegfaR1 and Nrp1 receptors at different stages of development. (*F*) Immunostainings for VegfaR1, Nrp1 and Nrp2 receptors (red) in dissociated cultured E14.5 Gad67-Gfp+ interneurons. (*G*) RT-PCR analysis of *VegfaR1*, *Nrp1*, *Nrp2* and *Gapdh* transcripts in FACS-isolated E13.5 Gad67-Gfp+ interneurons from the MGE and cortex (Cx). (Str, striatum; cpl, choroid plexus; MZ, marginal zone; CP, cortical plate; SP, subplate; IZ, intermediate zone; LIZ/SVZ, lower intermediate zone/subventricular zone; VZ, ventricular zone).

To investigate if *Vegfa* signaling influences interneuron development, we first examined its expression in relation to these cells. At E13.5, corresponding to early stages of interneuron migration, *Vegfa mRNA* was robustly localized within the MGE, where these cells are born (Fig. 1*A*). In addition, interneurons migrating along the deep migratory stream at the level of the subventricular/lower intermediate zone (SVZ/LIZ) were located close to *Vegfa* expression in the dorsal ventricular zone (VZ), with the superficial migratory stream located below the *Vegfa*-expressing meninges (Fig. 1*A*). Thus, both MGE progenitors and migrating interneurons are located close to sources of *Vegfa* at early phases of their migration, an association maintained at mid (E14.5) (data not shown) and late (E17.5) phases (Supplementary Fig. 1*A*). Blood vessels were recently suggested to be a source of *Vegfa* and to influence cortical interneuron migration in vitro*.* To assess whether blood vessels in the meninges expressed Vegfa, we labeled the microvasculature with IB4 together with the pericyte-specific Cd140b protein and Vegfa protein. This showed that IB4^+^Cd140b^**−**^ vascular endothelial cells (VECs) also expressed Vegfa (Fig. 1*B* and Supplementary Fig. 1B), as clearly observed in the PVP at E13.5 (Fig. 1*B*). Thus, these data show that MGE progenitors and blood vessels are sources of Vegfa in the embryonic forebrain. We next assessed whether interneurons express cognate Vegfa receptors and can respond to Vegfa signaling. We found that transcript for the *Neuropilin 1* receptor (*Nrp1*) was expressed throughout the MGE, with ~40–50% of postmitotic Gad67-gfp^+^ interneurons expressing *Nrp1* throughout all stages of their migration and ~20% of cells expressing the tyrosine-kinase receptor VegfaR1 (Vieira et al. 2010) at early-, mid-, and late stages of their migration (Fig. 1*C,E*), but not VegfaR2 or VegfaR3 (Supplementary Fig. 2). The localization of VegfaR1 and Nrp1 at the cell soma and along the processes of E14.5 Gad67-Gfp^+^ interneurons, respectively, was further confirmed in immunostainings of cultured dissociated cells (Fig. 1*F*). In addition, we confirmed the presence of *VegfaR1* and *Nrp1* and *Nrp2* transcripts in FACS-isolated E13.5 Gad67-Gfp^+^ interneurons by RT-PCR analysis (Fig. 1*G*). Thus, our expression analysis is consistent with a proportion of interneurons directly responding to Vegfa signaling through Nrp1 and VegfR1.

**Figure 2.**
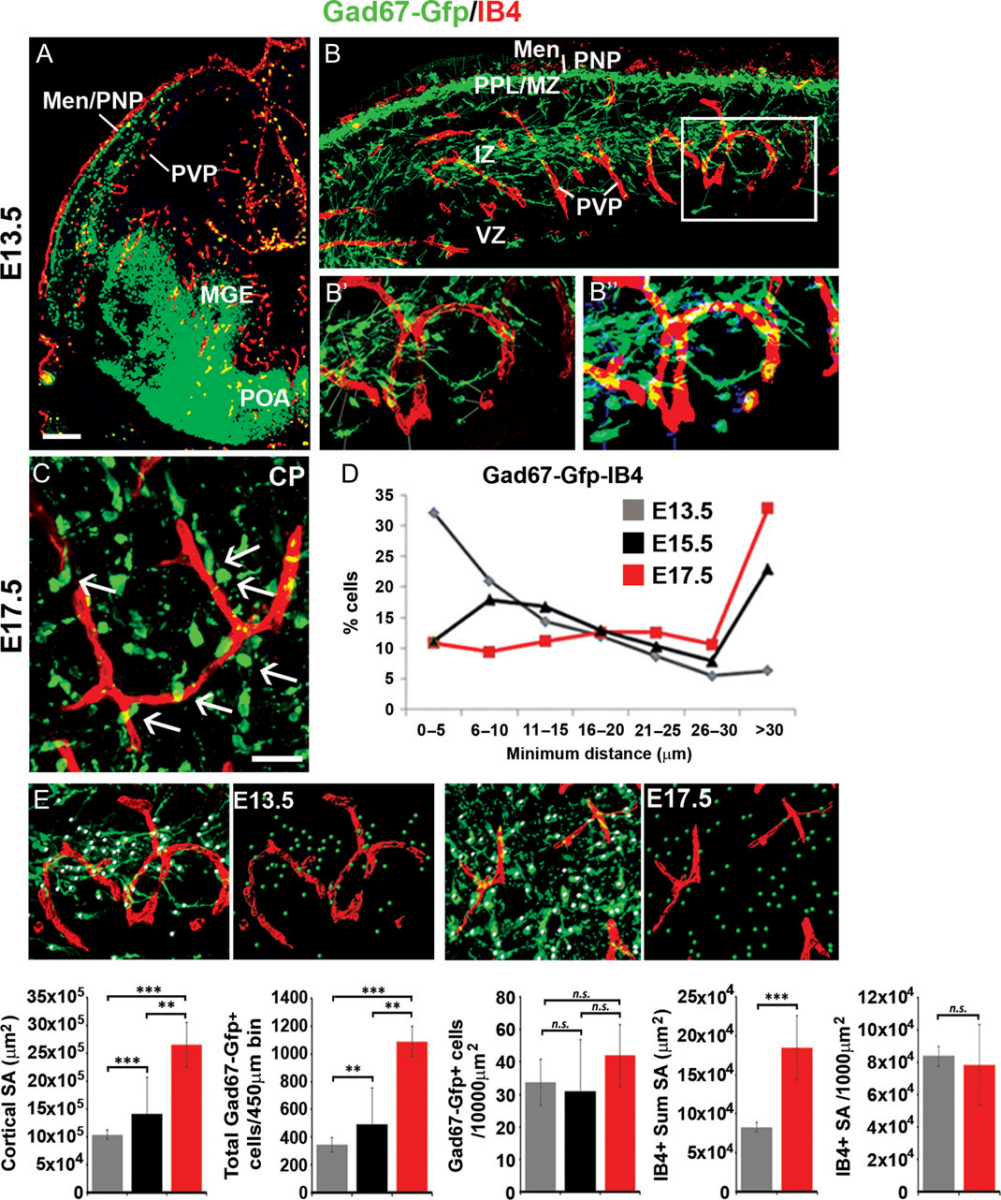
Interneurons are located in close proximity to blood vessels during their tangential and radial phases of migration. (*A*) Labeling of IB4^+^ blood vessels, together with immunostaining for Gad67-gfp^+^ interneurons in the E13.5 forebrain; cortex shown at high magnification in panel (*B*). (*B’*–*B”*) Panels show the acquired confocal image (B’ left panel) with the corresponding false-colored image (*B”* right panel) showing cells recognized using the bespoke plugin by thresholding according to immunostaining signal intensity and predicted cell size and used to quantify minimum distances between the centroid of migrating interneurons and the closest IB4^+^ vascular surface. (*C*) Labeling of IB4^+^ blood vessels together with immunostaining for Gad67-gfp^+^ interneurons in the E17.5 mouse cortex, showing radially oriented interneurons close to blood vessels in the CP. *(D)* Graphs showing the distribution of minimum intracellular distances between Gad67-gfp^+^ interneurons with IB4^+^ blood vessels measured using the bespoke plugin in the E13.5 (gray), E15.5 (black) and E17.5 (red) cortex. (*E*) Panels show confocal images of the E13.5 and E17.5 cortex of Gad67-Gfp+ mouse forebrains with Gad67-gfp+ cells detected using the Imaris software spot module (green spots) and surface rendering of blood vessels (red surfaces) used to quantify interneuron and vascular density. Graphs from left-right show: changes in cortical surface area (μm^2^), total Gad67-gfp^+^ interneuron numbers counted in 450 μm cortical bins, interneuron numbers normalized for cortical surface area, IB4^+^ blood vessel sum surface area, and density of IB4^+^ blood vessels normalized for cortical surface area at different stages of development (*t*-test, *n* = 3 for each; **P ≤ 0.01, ***P ≤ 0.001). (Men/PNP, Meninges/perineural plexus; PVP, periventricular plexus; MGE, medial ganglionic eminence; POA, preoptic area; MZ, marginal zone; CP, cortical plate; SP, subplate; IZ, intermediate zone; LIZ/SVZ, lower intermediate zone/subventricular zone; VZ, ventricular zone).

Our observations that blood vessels express Vegfa prompted us to re-examine their association with all GABA-synthesizing *Gad67-gfp*^*+*^ interneurons (Tamamaki et al. 2003) in the embryonic forebrain. At E13.5, a lattice-like IB4^+^ PVP network was positioned close to the MGE where most interneurons are born (Lavdas et al. 1999; Butt et al. 2005) (Fig. 2*A*). In addition, interneurons in the superficial and deep tangential streams migrated close to the respective PNP and PVP (Fig. 2*B*). At E17.5, we found radially oriented interneurons juxtaposed to IB4^+^ blood vessels in the CP (Fig. 2*C*). To quantify the spatial proximity of interneurons to the vasculature, we used a bespoke plugin to measure the minimum distance between the centroid of migrating cortical interneurons to the closest vascular surface in the developing cortex. Confocal-acquired images of immunostained forebrain sections (Fig. 2*B’*) were thresholded according to VEC and interneuron predicted cell size and signal intensity using an automated plugin, and the minimum distance from the centroid of interneurons to the closest vascular surface measured (Fig. 2*B”*). At E13.5, >90% of interneurons were located ≤30 μm from the closest vascular surface, compared with ~10% at E15.5–17.5 (Fig. 2*D*). To assess whether increased distance of interneurons from blood vessels at late stages could be secondary to their decreased density which could occur as the growing cortex expands over time, we quantified total interneuron numbers in the dorsal cortex at E13.5, E15.5, and E17.5, and normalized these numbers for changes in cortical surface area. This showed that while there was over a ~250% increase in cortical surface area between E13.5 to E18.5, the total number of cortical interneurons increased by more than ~300%, with no significant changes in interneuron density observed within the dorsal cortex over this time (Fig. 2*E*). Analysis of IB4^+^ blood vessels at E13.5 and E18.5 showed a significant ~220% increase in their sum surface area, with no significant changes in vascular density when normalizing for cortical surface area over this time. Together, this suggests that a greater proportion of interneurons are positioned close to blood vessels at early, compared with late, stages of development and that this is not due to their altered densities. Thus, consistent with prior studies, we found that interneurons migrate in close proximity to blood vessels during their tangential- (Won et al. 2013), but also during their radial phases of migration, raising the possibility that they may respond to vascular-Vegfa sources.

### Perturbing VEGF Signaling Through Ubiquitous Expression of Vegfa120 Alters Cortical Interneuron Numbers

**Figure 3.**
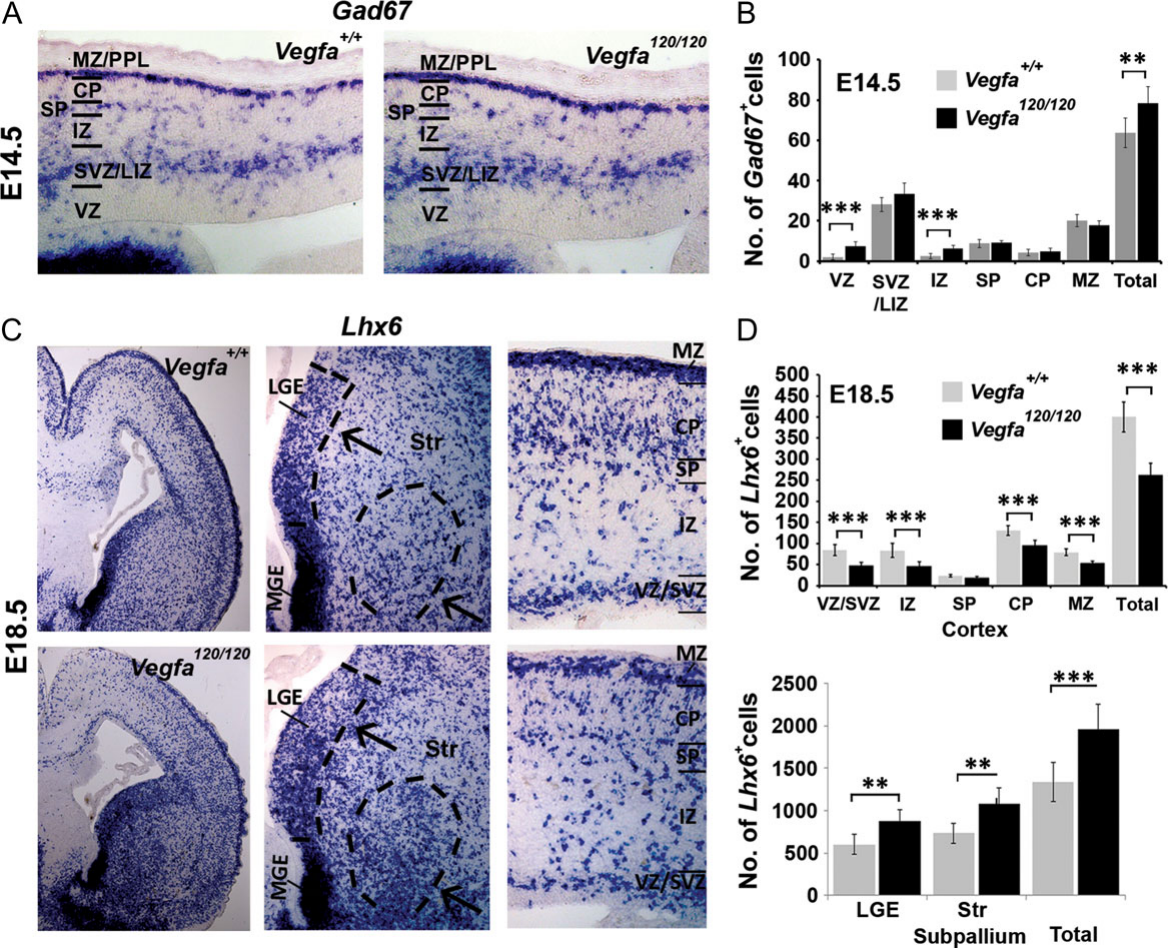
Altered cortical interneuron numbers at mid and late stages of cortical development in the *Vegfa*^*120/120*^ mutant forebrain. (*A*) In situ hybridization for the interneuron-marker *Gad67* in the cortex of E14.5 *Vegfa*^*+/+*^ and *Vegfa*^*120/120*^ mouse forebrains. (*B*) Quantification of interneuron number at E14.5. Data represented as mean values ± SEM (**P ≤ 0.01, ***P ≤ 0.001; *t*-test; *n* = 3 for each). (*C*) In situ hybridization for the interneuron-specific *Lhx6* mRNA in the cortex of E18.5 *Vegfa*^*+/+*^ and *Vegfa*^*120/120*^ mouse forebrains with dotted lines delineating the LGE VZ/SVZ and the ventral striatum in high magnification images of the subpallium, corresponding to regions where an ectopic increase in *Lhx6*^*+*^ interneurons is observed, and with a reduction in interneurons observed in high magnification of the dorsal cortex. (*D*) Bar charts show mean total number of *Lhx6*^*+*^ interneurons quantified in cortical regions and within the subpallium at E18.5 (*t*-test, *n* = 3 for each; **P ≤ 0.01, ***P ≤ 0.001).

To address whether Vegfa signaling plays a role in cortical interneuron development in vivo, we next analyzed their migration in transgenic knock-in mice which ubiquitously express the diffusible *Vegfa120* (*Vegfa*^*120/120*^) isoform, and which lack the heparin-binding *Vegfa165* and *Vegfa188* isoforms (Carmeliet et al. 1999; Ferrara 2010). This circumvented early-embryonic lethality which occurs upon depletion of all Vegfa isoforms, and enabled us to analyze interneuron development when the Vegfa chemotactic gradient was perturbed and the prevalent *Vegfa165* isoform was lacking. Cell counts at E14.5 showed a significant ~19% increase in total Gad67^+^ interneurons in the cortex of *Vegfa*^*120/120*^ mutants as well as a subtle shift in their distribution away from the superficial MZ and towards the deeper cortical layers (Fig. 3*A*,*B*; Supplementary Fig. 3*A*). In contrast, analysis at E18.5, corresponding to a time when interneurons have completed their tangential migration, but continue to migrate radially within the cortex, showed a striking ~41.4% decrease in total *Gad67*^*+*^ cells and a 34.5% decrease in MGE-derived *Lhx6*^*+*^ interneurons throughout most of the cortical thickness (Fig. 3*C,D*; Supplementary Fig. 3*B*). A concomitant increased accumulation of *Lhx6*^*+*^ and *Gad67*^*+*^ interneurons was also apparent within the SVZ of the subpallial LGE and the ventral striatum, corresponding to regions through which MGE-derived interneurons migrate *en route* to the cortex (Marin et al. 2001), suggesting that they follow their characteristic rostral-dorsal trajectories towards the cortex, but stall within the subpallium. As Vegfa promotes cortical and hippocampal neural progenitor proliferation in vivo (Darland et al. 2011; Fournier et al. 2012), we asked whether altered MGE progenitor proliferation could account for the altered interneuron numbers in the *Vegfa*^*120/120*^ cortex, but found no changes in PH3^+^ cells within the germinal VZ of the mutant MGE or in in vitro proliferation assays (Supplementary Fig. 4*A,B*). Vegfa has also been shown to promote neuronal survival during development (Cariboni et al. 2011), and so we could not exclude the possibility that cortical interneurons had migrated correctly, but were not viable. However, assessing for cell death, using the apoptotic marker cleaved caspase-3, showed its expression in IB4^+^ blood vessels, but not in interneurons (Supplementary Fig. 4*C*). An in vitro assay further showed that Vegfa120 and Vegfa165 isoforms had no effect on interneuron viability (Supplementary Fig. 4*D*). Thus, the aberrant accumulation of interneurons in the *Vegfa*^*120/120*^ subpallium and their concomitant decrease in the cortex, in the absence of evident cell death or altered proliferation, suggested that ubiquitous expression of Vegfa120 promotes MGE-derived interneuron migration at mid phases, but results in their defective migration at late stages of development.

### Increased Interneuron Numbers in the Cortex of Vegfa^120/120^ Mutants with a Defective Vascular Network at Mid Stages of Development

**Figure 4.**
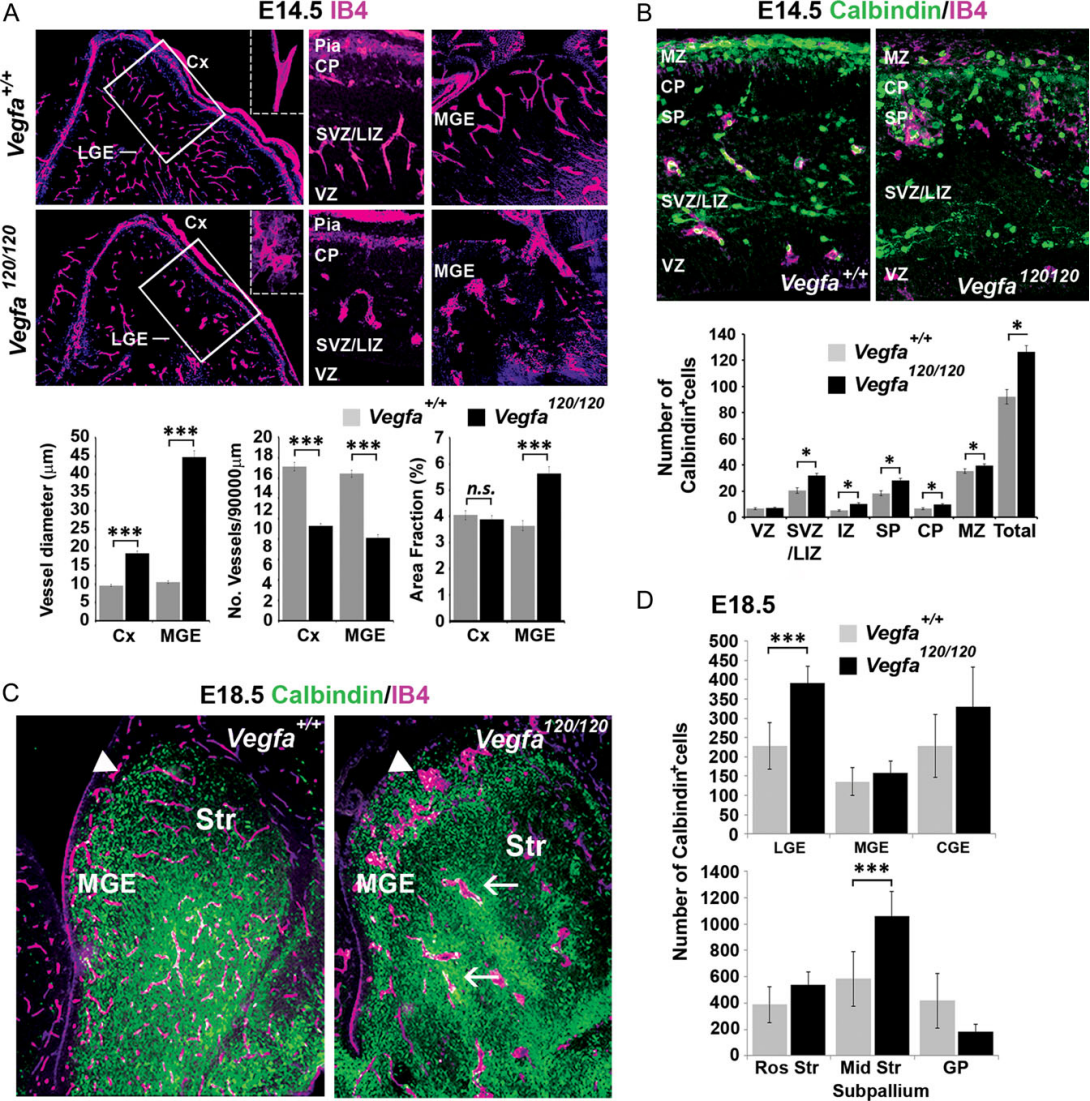
Interneurons tangential migration to the cortex is impeded at late but not mid phases of development in the *Vegfa*^*120/120*^ mutant forebrain with a defective vascular network. (*A*) IB4^+^ labeling of blood vessels in coronal sections of E14.5 *Vegfa*^*+/+*^ wildtype and *Vegfa*^*120/120*^ knock-in mouse forebrain; with vessel diameter, number and area fraction occupancy quantified in bar charts. (***P ≤ 0.0001, *t*-test; *n* = 4 for each). (*B*) IB4^+^ labeling of blood vessels and immunolocalization of Calbindin^+^ interneurons in coronal sections of E14.5 *Vegfa*^*+/+*^ wildtype and *Vegfa*^*120/120*^ knock-in mouse forebrain, with mean number of interneurons represented in bar charts. (*P ≤ 0.05; *t*-test; *n* = 3 for each). (*C*) IB4^+^ labeling of blood vessels and immunolocalization of Calbindin^+^ interneurons in the coronal sections through the subpallium of E18.5 *VegfA*^*+/+*^ and *VegfA*^*120/120*^ mouse forebrains. Arrows show accumulation of interneurons adjacent to dilated blood vessels. (Data in all graphs represent values ±SEM). (*D*) Mean number of Calbindin^+^ cells quantified in subpallial regions shown by bar charts. LGE, lateral ganglionic eminence; Cx, cortex; CP, cortical plate; SP, subplate; IZ, intermediate zone; IZ/SVZ, lower intermediate zone/subventricular zone; VZ, ventricular zone.

Previous studies have shown that the correct ratio of Vegfa isoforms is required to form an extracellular chemotactic gradient along which blood vessels grow in the developing brain (Ruhrberg et al. 2002). A recent study which induced meningeal cell death to perturb the PNP or an anti-angiogenic inhibitor to perturb the PVP in cortical slices *ex-vivo* showed that this disrupted the formation of the tangential migratory streams of interneurons (Won et al. 2013). This study further showed that PVP and PNP VECs secrete chemoattractive factors which promote the movement of interneurons isolated from the MGE and superficial migratory stream. However, whether the vasculature guides migrating interneurons in vivo in the intact forebrain remains unknown. Thus, we asked whether a defective vascular network could be responsible for the altered cortical interneuron numbers in *Vegfa*^*120/120*^ mutants. Labeling of the vasculature in E14.5 *Vegfa*^*120/120*^ mutants with IB4 showed a significantly decreased number and increased size of IB4^+^ particles in the *Vegfa*^*120/120*^ forebrains indicative of their aberrant dilated vessels, which was especially prevalent in the subpallium (Fig. 4*A*). We next examined the number and distribution of Calbindin^+^ interneurons relative to the defective vascular network. As shown with previous interneuron markers, a significant, albeit more striking, 37% increase in Calbindin^+^ interneurons was observed in the cortex of E14.5 *Vegfa*^*120/120*^ mutants with defective blood vessels (Fig. 4*B*). Analysis at E18.5 showed that the vascular defect persisted and included the LGE SVZ and ventral striatum where an increased accumulation in Calbindin^+^ interneurons was observed (Fig. 4*C,D*). Interestingly, Calbindin^+^ interneurons were sometimes clustered around dilated blood vessels in the subpallium. This suggested that late, but not early, migrating interneurons may require an intact vascular network to reach the dorsal cortex or, alternatively, that they may differentially respond to a chemotropic source of Vegfa120.

### VEGF Isoforms Differentially Promote the Migration of Early- and Late-Migrating Interneurons In Vitro

**Figure 5.**
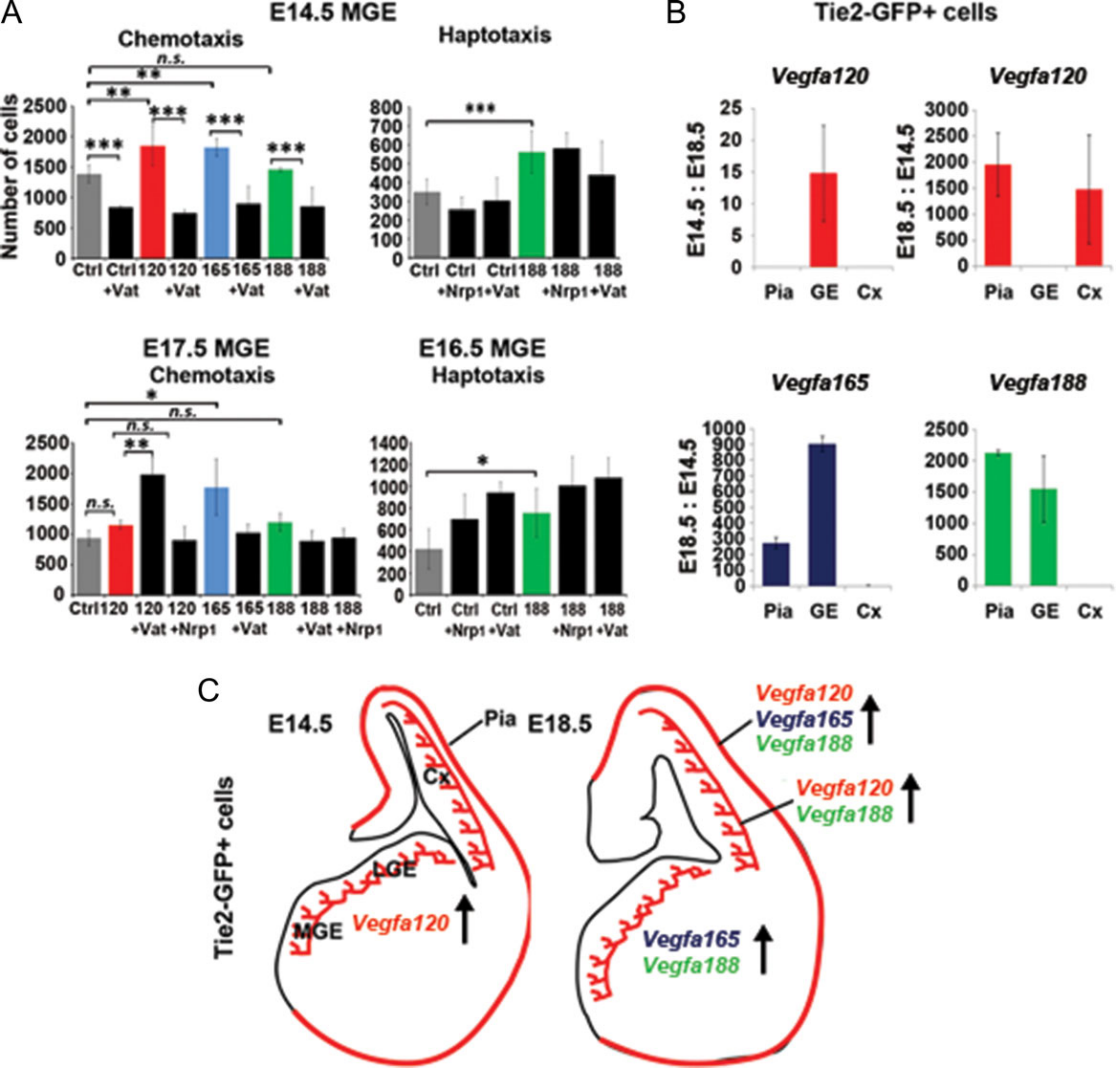
Vegfa120 isoforms positively regulate the migration of early MGE-derived interneurons, with Vegfa165 and Vegfa188 promoting the migration of later MGE-derived cells in vitro, at a time when cortical Tie2+ VECs upregulate *Vegfa165* and *Vegfa188 *in vivo. (*A*) Quantification of the number of E14.5 and E17.5 MGE cells migrating in response to recombinant Vegfa120, Vegfa165 and Vegfa188 proteins, in the presence/absence of the tyrosine-kinase VegfaR1-inhibitor Vatalanib (Vat) and a Nrp1 blocking antibody (Nrp1), assayed in Boyden chemotaxic and haptoptactic in vitro assays (*t*-test, *n* = 3 for each; *P ≤ 0.05, **P ≤ 0.01, ***P ≤ 0.001). (*B*) qPCR analysis showing fold change in *Vegfa* isoform-specific gene expression levels in Tie2-Gfp^+^ cells FACS-isolated from the pia, ganglionic eminence (GE) and cortex (Cx) of E14.5 or E18.5 Tie2-Gfp+ mouse forebrains. Charts compare the ratio of gene expression levels between E14.5 and E18.5 and E18.5 and E14.5 for Tie2-Gfp^+^ cells isolated from corresponding forebrain regions (*n* = 3). (*C*) Schemas summarizing qPCR analysis depicting relative changes in *Vegfa* isoforms expressed by Tie2+ VECs at each developmental stage.

To distinguish between these possibilities, we directly tested whether Vegfa influences interneuron migration, using the Boyden chemotaxic and modified haptotactic assays to assess the response of MGE-derived cells to recombinant Vegfa isoforms. This showed that E14.5 MGE cell migration was enhanced by all Vegfa isoforms, a response attenuated by the tyrosine-kinase VegfaR1-2 inhibitor Vatalanib, while later-born E16.5/17.5 MGE cell migration was enhanced only by Vegfa165 and Vegfa188 isoforms (Fig. 5*A*). Thus, Vegfa isoforms differentially regulate early and late MGE-derived migrating interneurons in vitro*.* This was consistent with the altered interneuron numbers reaching the dorsal cortex in the *Vegfa*^*120/120*^ mutants and suggested that cortical interneurons require Vegfa165 and Vegfa188 isoforms for their correct tangential migration at late stages of development. Vegfa splicing occurs in a temporal and tissue-specific manner in the developing brain (Darland et al. 2011; Cain et al. 2014). We, thus, next asked whether the endogenous vasculature express Vegfa heparin-binding isoforms in vivo at late stages of interneuron migration. To determine this, we FACS- purified Tie2^+^ VECs isolated from the meninges, dorsal cortex and subpallium at mid (E14.5) and late stages (E18.5) of interneuron migration. This showed that *Vegfa120* was expressed most strongly in the GE at mid stages and within the cortex at late stages, at a time when the heparin-binding *Vegfa165* and *Vegfa188* isoforms were also upregulated in the pia (Fig. 5*B,C*). Thus, these data show that the endogenous microvasculature express *Vegfa* isoforms in a regional and temporal manner, and upregulate *Vegfa165* and *Vegfa188* at late phases of migration. Together, this confirmed a chemotropic role for Vegfa in regulating cortical interneuron migration and raised the possibility that vascular-Vegfa may be involved.

### Endothelial Depletion of Vegfa Reduces Cortical Interneuron Numbers, Decreases Their Proximity to Blood Vessels and Alters Their Positioning in the CP

**Figure 6.**
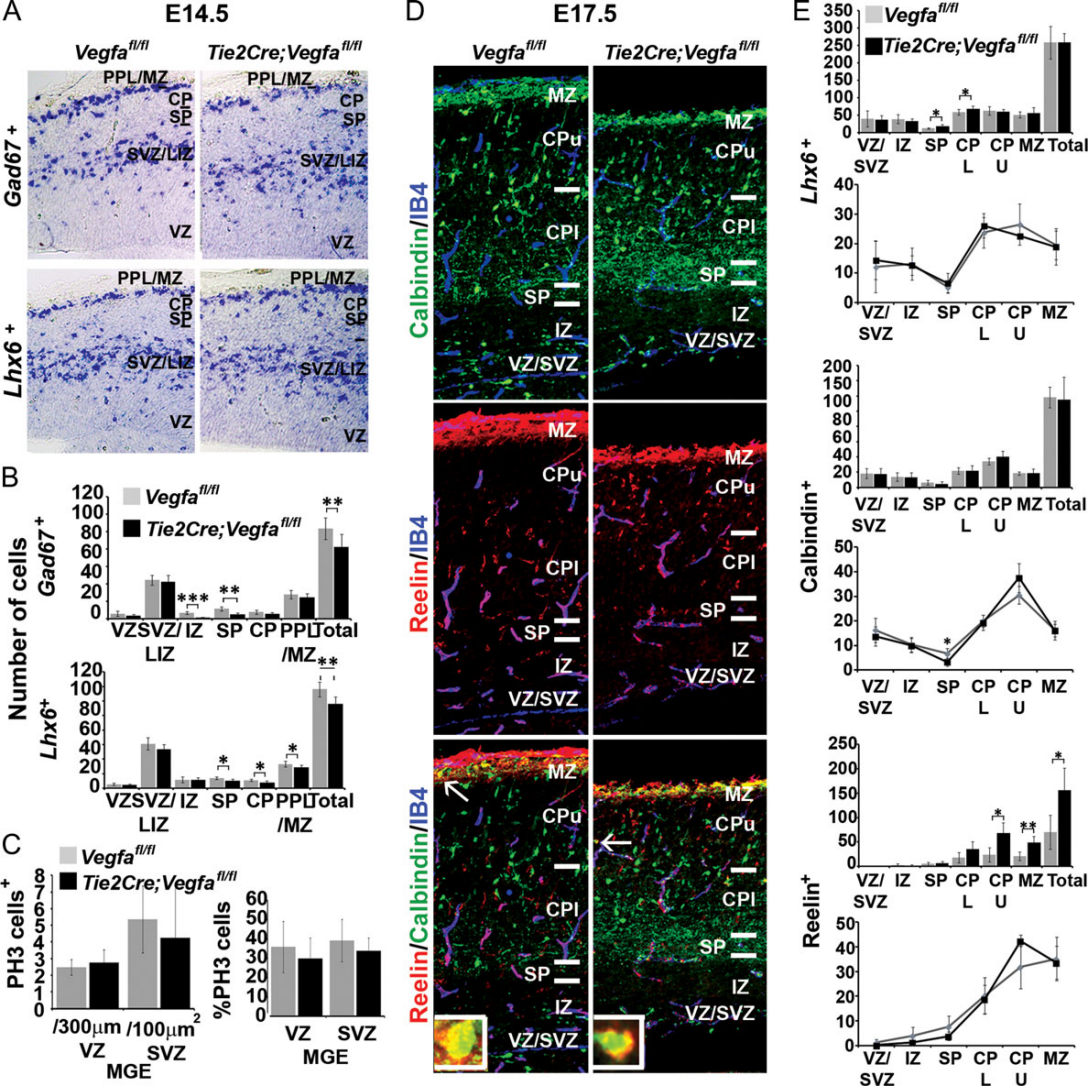
Decreased cortical interneuron numbers and alterations in their intracortical distribution in *Tie2Cre;Vegfa*^*fl/fl*^ mutant forebrains. (*A*) In situ hybridization for the *Gad67* and *Lhx6* mRNA in the cortex of E13.5 *Vegfa*^*fl/fl*^ control (gray) and *Tie2Cre;Vegfa*^*fl/fl*^ mutant (black) and their (*B*) quantifications in cortical regions (Student’s *t*-test; *n* = 4 when combining *n* = 2 at E13.5 combined with *n* = 2 E14.5; *P ≤ 0.05, **P ≤ 0.01, ***P ≤ 0.001). (*C*) Graphs show mean total numbers and percentages of PH3^+^ proliferating MGE progenitors in the E13.5/E14.5 *Vegfa*^*fl/fl*^ control and *Tie2Cre;Vegfa*^*fl/fl*^ mutant animals (Student’s unpaired *t*-test, *n* = 4 when combining *n* = 2 at E13.5 combined with *n* = 2 E14.5; *P ≤ 0.05, **P ≤ 0.01, ***P ≤ 0.001). (*D*) Immunolabelling of Calbindin+ and Reelin+ cells and IB4^+^ blood vessels, in the cortex of E17.5 *Vegfa*^*fl/fl*^ and *Tie2Cre;Vegfa*^*fl/fl*^ mutant mice with (*E*) their total numbers and intracortical distributions represented graphically. (Student’s unpaired *t*-test, *n* = 3 for each; *P ≤ 0.05, **P ≤ 0.01) (Graphs show mean values ±SEM).

**Figure 7.**
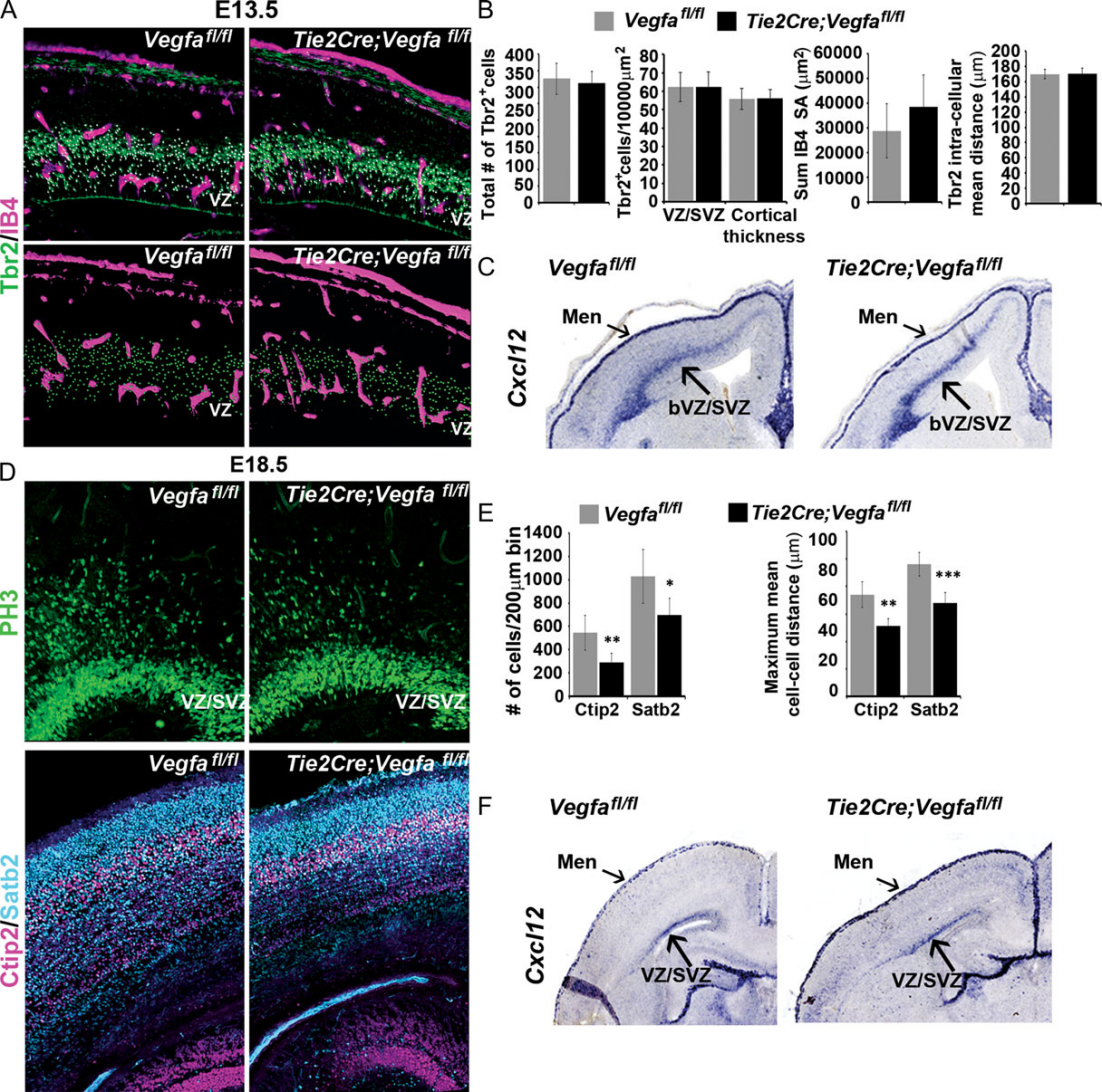
Unaltered number and density of intermediate progenitors or in their expression of Cxcl12/Sdf1 in E13.5 *Tie2Cre;Vegfa*^*fl/fl*^ mutant forebrains. (*A*) Images of the dorsal cortex of E13.5 *Vegfa*^*fl/fl*^ control and *Tie2Cre;Vegfa*^*fl/fl*^ mutant forebrains immunostained for Tbr2^+^ intermediate progenitor cells (IPCs) and with the vasculature labeled with isolectin-B4. Spots superimposed on Tbr2^+^ nuclei show automated detection of Tbr2^+^ cells using Imaris software spot module function, with spots in bottom panels showing the distribution of Tbr2^+^ cells and surface rendering of the vasculature used in quantifications shown in bar charts. (*B*) Graphs show the total number (left) and density of Tbr2^+^ cells within the neurogenic basal VZ/SVZ and normalized for differences in cortical thickness in E13.5 *Vegfa*^*fl/fl*^ control and *Tie2Cre;Vegfa*^*fl/fl*^ mutant forebrains (second left). Graphs on right shows the surface area of IB4^+^ blood vessels in the cortex of E13.5 *Vegfa*^*fl/fl*^ control and *Tie2Cre;Vegfa*^*fl/fl*^ mutant measured from surface renderings generated with Imaris software and the mean intra-cellular distance of Tbr2+ cells. (*C*) In situ hybridization analysis of *Cxcl12/Sdf1* transcripts in the E13.5 *Vegfa*^*fl/fl*^ control and *Tie2Cre;Vegfa*^*fl/fl*^ mutant forebrains. (*D*) Immunolabelling of Tbr2^+^ IPs (top panels), Ctip2^+^ and Satb2^+^ pyramidal neurons (bottom panels) in the dorsal cortex of E17.5 *Vegfa*^*fl/fl*^ control and *Tie2Cre;Vegfa*^*fl/fl*^ mutant forebrains with (*E*) bar charts showing the mean total numbers and intercellular distances of Ctip2^+^ and Satb2^+^ cells. (*F*) Expression of *Cxcl12/Sdf1* transcripts in the E17.5 *Vegfa*^*fl/fl*^ control and *Tie2Cre;Vegfa*^*fl/fl*^ mutant forebrains (*t*-test, *P ≤ 0.05, **P ≤ 0.01, ***P ≤ 0.001) (Bar graphs show mean values ±SEM).

To investigate whether interneurons respond to vascular-derived *Vegfa *in vivo, we used the floxed *Vegfa* mouse line *(Vegfa*^*fl/fl*^) to conditionally ablate all isoforms upon Cre-mediated recombination within the Tie2^+^ lineage (Kisanuki et al. 2001), which efficiently targets all blood vessels in the embryonic mouse forebrain (*Tie2Cre;Vegfa*^*fl/fl*^). This showed a significant ~20% decrease in total *Gad67*^*+*^ and *Lhx6*^*+*^ interneurons in the E14.5 *Tie2Cre;Vegfa*^*fl/fl*^ mutant cortex which occurred in the absence of altered MGE progenitor proliferation, as assessed by PH3^+^, or in the positioning of PH3^+^ progenitors relative to the IB4^+^ microvasculature (Fig. 6*A–C*). Further, CC3^+^ cells rarely colocalised with interneuron markers suggesting that their survival was not compromised (data not shown). Analysis at late stages showed that the cortex was markedly thinner, consistent with reported deficits in the generation of pyramidal neurons upon perturbation of Vegfa signaling (Darland et al. 2011; Cain et al. 2014). Surprisingly, no differences in interneuron numbers were found in the cortex at this time, although there were subtle changes in the distribution of Calbindin^+^ and Reelin^+^ interneurons in the CP (Fig. 6*D,E*). As Vegfa has been shown to directly modulate neurogenesis in the dorsal cortex (Haigh et al. 2003; Darland et al. 2011; Cain et al. 2014), and cortical intermediate progenitors (IPs), in turn, shown to promote the tangential migration of interneurons through their secretion of the chemokine stromal-derived factor-1 (Sdf1/Cxcl12) (Tiveron et al. 2006; Sessa et al. 2010; Abe et al. 2015), we next asked whether depletion of Vegfa from blood vessels could indirectly influence interneuron migration through its action on IPs. We, thus, assessed for the IP marker, T-box brain-2 transcription factor (Tbr2) (Englund et al. 2005; Kowalczyk et al. 2009), by immunohistochemistry in the E13.5 *Tie2Cre;Vegfa*^*fl/fl*^ mutant and *Vegfa*^*fl/fl*^ control cortex, together with IB4^+^ labeling of the vasculature. This showed there were no differences in the total number or density of Tbr2^+^ cells within the neurogenic basal VZ/SVZ, or when Tbr2^+^ counts were normalized for potential differences in cortical thickness (Fig. 7*A,B*). Tbr2^+^ cells have been shown to be intimately associated with blood vessels (Javaherian and Kriegstein 2009; Stubbs et al. 2009) and autocrine-Vegfa signaling has been shown to be important for the survival of blood vessels in the adult cortex (Lee et al. 2007), so we next asked whether blood vessels were altered upon endothelial ablation of Vegfa. This showed no changes in vascular surface area in the *Tie2Cre;Vegfa*^*fl/fl*^ mutant and *Vegfa*^*fl/fl*^ control cortex or in the intercellular distances between Tbr2^+^ cells, suggesting no changes in IPs distribution within the VZ/SVZ (Fig. 7*B*). We next asked whether IPs expression of *Sdf1*, required to promote the tangential migration of interneurons, was altered. In situ hybridization analysis showed that *Sdf1* transcripts were expressed at similar levels and correctly localized within the basal VZ/SVZ and meninges of the dorsal cortex in the *Tie2Cre;Vegfa*^*fl/fl*^ mutant and *Vegfa*^*fl/fl*^ control animals at E13.5 (Fig. 7*C*). Analysis at E18.5, however, suggested a possible decrease in the number of Tbr2^+^ IPs, together with decreased number of Ctip2^+^ and Satb2^+^ cells, corresponding to prospective layer IV and II–IV pyramidal neurons (Alcamo et al. 2008; Britanova et al. 2008; Woodworth et al. 2016) (Fig. 7*D,F*) suggesting that vascular depletion of Vegfa results in defective neurogenesis in the dorsal cortex at late stages. Interestingly, however, there was no obvious change in the expression of Sdf1 in the VZ/SVZ or meninges of *Tie2Cre;Vegfa*^*fl/fl*^ mutant and *Vegfa*^*fl/fl*^ control cortex at this time. Together, this shows that the observed reduction in cortical interneuron numbers at mid stages of corticogenesis upon endothelial ablation of Vegfa (Fig. 6*A-B*) occurs in the absence of defects in IPs or in their expression of Sdf1 (Fig. 7*A–C*), consistent with vascular-Vegfa directly promoting interneuron migration at early-mid stages of development.

**Figure 8.**
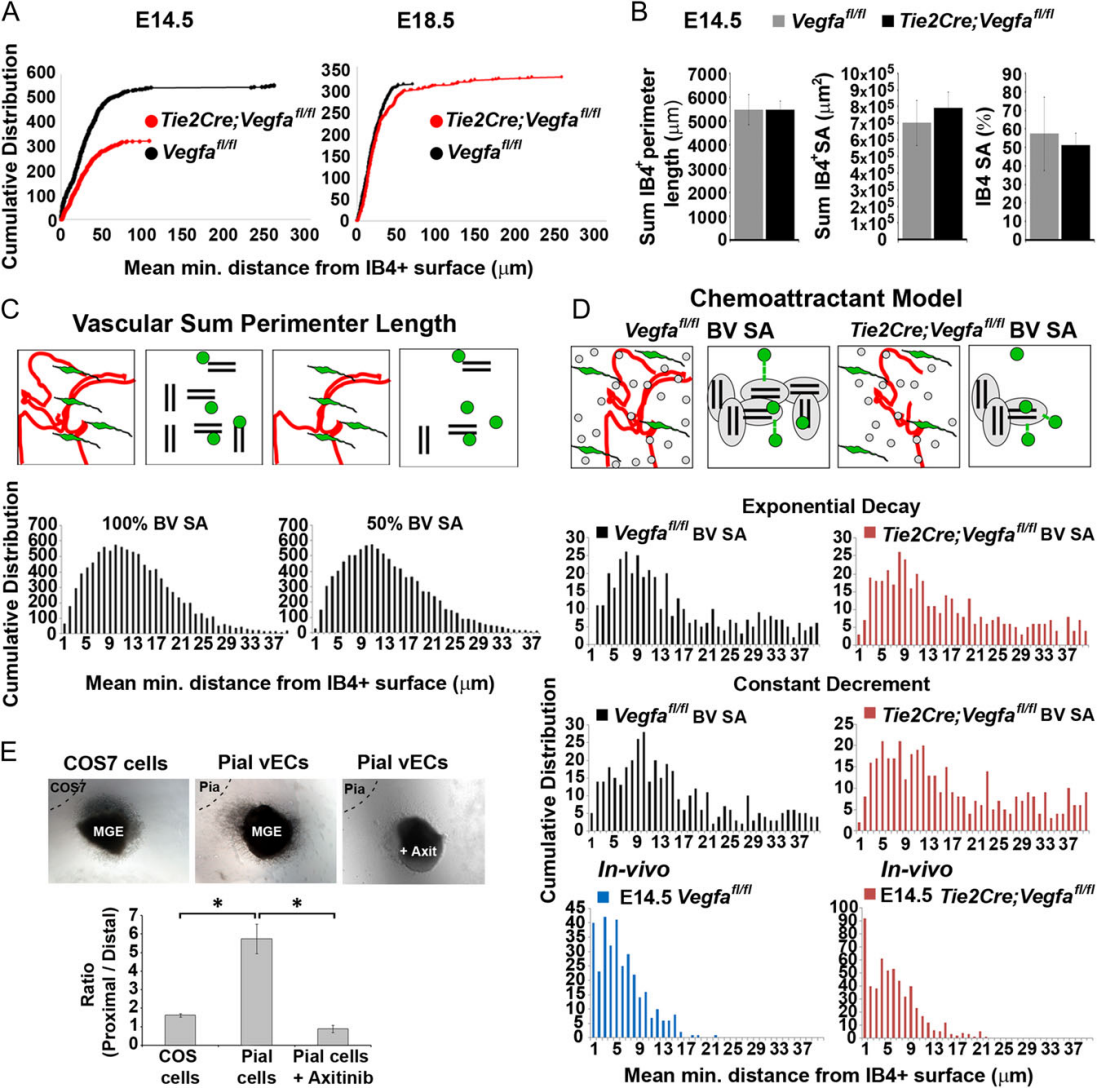
Decreased proximity of migrating cortical interneurons with the vasculature upon endothelial depletion of *Vegfa* (*A*) Cumulative distribution showing all measurements of mean minimum distances between the centroid of Calbindin^+^ interneurons and the closest IB4^+^ vascular surface in E14.5 and E18.5 *Vegfa*^*fl/fl*^ control and *Tie2Cre;Vegfa*^*fl/fl*^ mutant cortex (E14.5, *Vegfa*^*fl/fl*^*n* = 368 cells, *Tie2Cre;Vegfa*^*fl/fl*^, *n* = 476 cells; E18.5, *Vegfa*^*fl/fl*^*n* = 311 cells, *Tie2Cre;Vegfa*^*fl/fl*^, *n* = 327 cells) (KS-test, *P* ≤ 0.001 for all, maximum difference between cumulative distributions (D) at E13.5, D is 0.1294; E14.5, D is 0.1470; E18.5, D is 0.1698). (*B*) Automated measurements of the sum perimeter lengths, surface area and % surface area occupancy of IB4^+^ periventricular (PVP) and perineural (PNP) vascular plexi in the E14.5 *Vegfa*^*fl/fl*^ and *Tie2Cre;Vegfa*^*fl/fl*^ cortex. (*C*) Schematics show how the in vivo representations of blood vessels and interneurons (left panels) were modeled by pairs of lines and spots, respectively (right panels). Both objects were randomly distributed in a 3D volume space and the mean minimum intra-object distances calculated in the same manner as when analyzing the in vivo interneuron-vascular distributions. Graphs show how changing blood vessel (BV) surface area (SA) influences the relative distributions of interneurons with vascular surfaces when these are randomly distributed. (*D*) Schematic representation of in vivo and simulated representations of blood vessels (red lines) and interneurons (green cells) in vascular-chemoattraction models; in which chemoattraction was modeled as an exponential decay or as a constant decrement. Graphs show mean minimum distances measured between simulated interneurons (spots) and blood vessels (lines) in vascular chemoattraction exponential decay or constant decrement models. Magenta bar charts show the same simulations in which the vascular surface area is reduced by 30% to simulate the decreased vascular surface area observed in the *Tie2Cre;Vegfa*^*fl/fl*^ in vivo. Bottom bar charts show in vivo mean minimum distances measured between the centroid of Calbindin^+^ interneurons with the closest IB4^+^ vascular surface in the *Vegfa*^*fl/fl*^ animals (blue) and *Tie2Cre;Vegfa*^*fl/fl*^ mutants (magenta) using the bespoke plugin. (*E*) E15.5 rat MGE explants cocultured with cortical pial explants dissected from the same forebrain, or with control COS cells, in the presence/absence of the VegfR1-R3 inhibitor Axitinib (*n* = 3 experiments) (*t*-test, *P ≤ 0.05) (Bar graphs show mean values ±SEM).

We next posited that if interneurons respond to a chemotropic source of vascular-Vegfa, this could influence their distribution relative to the vasculature. Measurements of the minimum distance of Calbindin^+^ cells relative to the IB4^+^ blood vessels showed that they were positioned further away from Vegfa-ablated cortical blood vessels at all stages (Fig. 8*A*), despite an unaltered number of Calbindin^+^ interneurons (Supplementary Fig. 5*A*) and with no significant differences in vascular surface area (Fig. 8*B* and Supplementary Fig. 5A). As Vegfa directly impacts both interneurons and the vasculature, we asked whether the association of interneurons with blood vessels could be due to their random distributions alone. We thus generated models of the relative distribution of 2 randomly positioned objects (cell types) which showed that this resulted in a Gaussian distribution of intra-object minimum distances, and failed to recapitulate the in vivo distributions in either *Vegfa*^*fl/fl*^ control or *Tie2Cre;Vegfa*^*fl/fl*^ mutant animals. Their Gaussian distributions were maintained even when we changed parameters of vascular surface area or area fraction occupancy to assess whether increasing vascular surface area increases the probability that randomly positioned interneurons are localized closer to blood vessels, suggesting that their proximity to the vasculature required additional factors (Fig. 8*C* and Supplementary Fig. 5*B-D*). We, thus, generated simple chemoattraction models in which 2 objects were randomly distributed, but the final positions of “interneurons” were determined by vascular-chemoattractant gradients to simulate migration, and found these more closely recapitulated the in vivo distributions (Fig. 8*D*).

To further explore this in vivo, we analyzed transgenic knock-in mice which ubiquitously expressed the heparin-binding Vegfa165 isoform (Carmeliet et al. 1999), and in which interneuron numbers and vascular morphogenesis was not compromised (Supplementary Fig. 6*A*). This showed that interneurons were positioned significantly closer to blood vessels in the E14.5 *Vegfa*^*165/165*^ cortex relative to wildtypes, in the absence of changes to the vasculature and in the absence of changes in the total number of Calbindin^+^ and *Lhx6*^*+*^ cortical interneurons (Supplementary Fig. 6*B*). This showed that the altered spatial proximities of interneurons to the vasculature was not secondary to an altered vascular surface area or increased interneuron numbers which would increase the probability of their proximity even if randomly distributed. Together, this supports our findings that a chemotropic source of vascular-Vegfa promotes interneuron migration and positioning and their spatial proximity to blood vessels.

To directly assess whether cortical interneurons are attracted by vascular-secreted cues, we carried out coculture experiments using VEC-enriched pial and MGE explants isolated from the embryonic rodent forebrain. This showed a robust increase in MGE cell migration towards pial-secreted factors which was attenuated by the VegfR1-3 tyrosine-kinase inhibitor Axitinib (Hu-Lowe et al., 2008) (Fig. 8*E*). This was consistent with early-migrating interneurons responding to Vegfa secreted by PNP-enriched pial cells, and with our in vivo data in which ablation of *Vegfa* from the Tie2^+^ microvasculature delayed interneuron migration and decreased their proximity to blood vessels, all together indicating that vascular-derived Vegfa affects positioning and migration of interneurons at early-mid stages of development.

**Figure 9.**
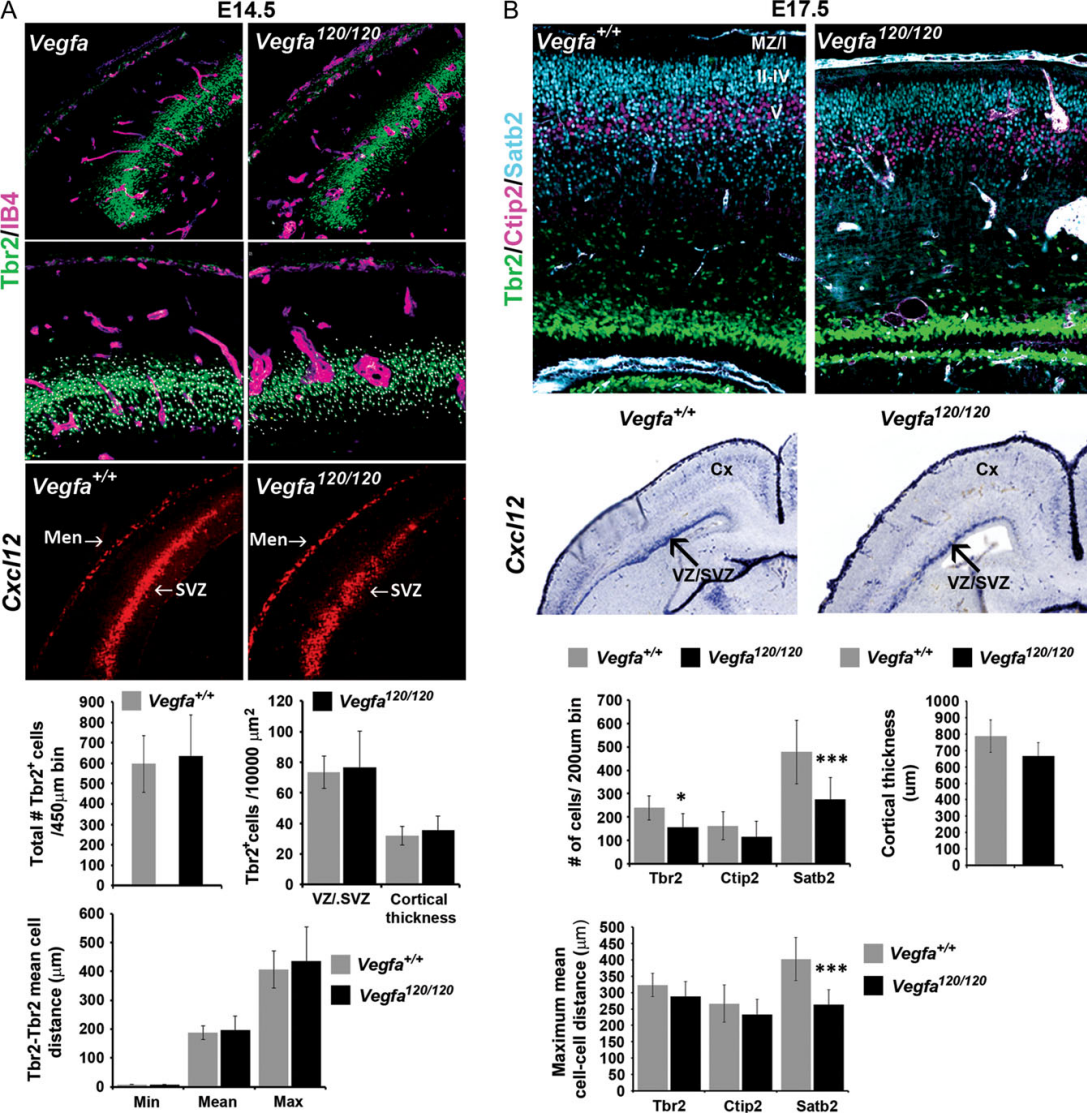
Defects in neurogenesis in the dorsal cortex of *Vegf*^*120/120*^ mutant forebrains at late stages of corticogenesis. (*A)* Immunodetection of Tbr2^+^ intermediate progenitors with Isolectin-B4^+^ labeling of blood vessels in the dorsal cortex of *Vegfa*^*+/+*^ control and *Vegfa*^*120/120*^ mutants at E14.5. Middle panels show high magnification of the dorsal cortex, with automated detection of Tbr2^+^ cells using the Imaris spot function module denoted by superimposed spots and used in quantifications of the total number and density of Tbr2^+^ cells in the cortex, and in their intracellular minimum, mean and maximum distances, represented graphically. Bottom panels show in situ hybridization for *Cxcl12/Sdf1* in the dorsal cortex of E14.5 *Vegfa*^*+/+*^ control and *Vegfa*^*120/120*^ mutants. Graphs represent the quantification of the total number, density and intercellular (minimum, mean, maximum) distance of Tbr2^+^ cells in the dorsal cortex of E14.5 *Vegfa*^*+/+*^ control and *Vegfa*^*120/120*^ mutants. (*B*) Immunolocalization of Tbr2^+^ (green), Ctip2^+^ (magenta) and Satb2^+^ (cyan) cells in the dorsal cortex of E17.5 *Vegfa*^*+/+*^ control and *Vegfa*^*120/120*^ mutant forebrains, with graphs showing the mean total number and intercellular distance of Tbr2^+^, Ctip2^+^ and Satb2^+^ cells quantified in the dorsal cortex. (*C*) In situ hybridization analysis of *Cxcl12/Sdf1* transcripts in the forebrain of E17.5 *Vegfa*^*+/+*^ control and *Vegfa*^*120/120*^ mutant animals. (*t*-test, *P ≤ 0.05, ***P ≤ 0.001) (Bar graphs show mean values ±SEM).

Our findings that endothelial ablation of Vegfa at late stages did not phenocopy the interneuron deficit observed in the *Vegfa*^*120/120*^ mutants showed that the tangential migration of interneurons at late phases was not dependent on vascular-Vegfa. We previously found that Vegfa was strongly expressed in the VZ/SVZ and forming CP of the dorsal cortex (Fig. 1*A-B*), raising the possibility that neural sources of Vegfa may influence interneuron migration either directly or indirectly through their expression of other factors such as *Sdf1*. We, therefore, assessed for potential changes in neurogenesis in the dorsal of *Vegfa*^*120/120*^ mutant and control forebrains at mid (E14.5) and late (E17.5) stages of corticogenesis. This showed that there were no changes in the total number or density of Tbr2^+^ IPs at E14.5 or in their expression of Sdf1 within the VZ/SVZ (Fig. 9*A*). Thus, the increased number of cortical interneurons observed at this stage is not likely due to defects in IPs or their expression of *Sdf1* and consistent with endothelial ablation of Vegfa promoting interneuron migration at this time. Analysis at E17.5, however, showed a significant decrease in the total numbers and density of Tbr2^+^ IPs in the *Vegfa*^*120/120*^ cortex compared with the control animals, as well as a reduction in Ctip2^+^ and Satb2^+^ cells, corresponding to prospective layer IV and II–IV pyramidal neurons (Fig. 9*B*). Together, this shows a reduction or delay in neurogenesis in the dorsal cortex in the *Vegfa*^*120/120*^ mutant forebrain at late stages of corticogenesis. As the number of IPs was significantly reduced at late stages, we next asked whether their expression of the chemokine *Sdf1* was affected. Surprisingly, we found no obvious reduction in the expression of Sdf1 at E17.5, with transcripts correctly localized in the SVZ and meninges of the *Vegfa*^*120/120*^ mutant dorsal cortex and comparable to the control animals (Fig. 9*C*). Our results raise the possibility that later-migrating interneurons may be impeded by neural-Vegfa and/or the reduced generation of IPs through other as of yet unidentified secreted factors. Together, these analyses support our findings that vascular-Vegfa and Vegfa isoforms promote interneuron migration to the dorsal cortex at early-mid stages of development, with neural-Vegfa likely playing a role at late stages of cortical development.

## Discussion

The intimate development of the vascular network and the nervous system is largely coordinated by Vegfa, a pro-angiogenic factor that acts on blood vessels, but which also has direct pleiotropic roles during neural development. Vegfa is expressed in multiple cell types, with signaling occurring in both a paracrine and autocrine manner. In the developing forebrain, Vegfa secreted by neural progenitors is essential for the correct and timely vascularization of the brain parenchyma at the onset of neurogenesis (Haigh et al. 2003; Raab et al. 2004), with autocrine-Vegfa required to maintain vascular homeostasis postnatally (Gerber et al. 1999; Lee et al. 2007). While Vegfa directly regulates neurogenesis in the adult hippocampus (Fournier et al. 2012) and neuronal migration in the hindbrain and spinal cord (Schwarz et al. 2004; Ruiz de Almodovar et al. 2010), its influence on these processes in the developing forebrain is less well elucidated. The biological relevance of distinct Vegfa isoforms in the early-embryonic forebrain was previously investigated by transcriptomic and immunohistochemical analyses of E9.5 and E11.5 *Vegfa*^*120/120*^, *Vegfa*^*188/188*^ and *Vegfa*^*120/188*^ mice, which suggested that Vegfa isoforms differentially regulate genes associated with neuronal proliferation, differentiation, patterning and migration (Darland et al. 2011; Cain et al. 2014). This further showed the altered expression of *Dlx1&2* in *Vegfa*^*188/188*^ and *Vegfa*^*188/120*^ knock-in mutants, which are essential homeodomain transcription factors required for the correct specification and migration of GABAergic interneurons. More recently, endothelial ablation of Vegfa was suggested to alter interneuron markers in the cortex; however, a detailed analysis or the specific mechanism underlying these changes was not addressed. Moreover, while vascular-secreted cues were previously suggested to guide migrating interneurons (Won et al. 2013), this was based on in vitro experiments, and evidence for blood vessels guiding interneurons in the intact forebrain was lacking.

Here, we asked whether Vegfa signaling influences cortical interneuron development in vivo by analyzing interneuron migration in transgenic mice which express a single Vegfa120 isoform to perturb the Vegfa extracellular gradient. Consistent with previous reports, we found that ubiquitous expression of Vegfa120 resulted in dramatically dilated blood vessels, likely due to aberrant proliferation and defective filopodial guidance of vascular endothelial tip-cells (Fig. 4A) (Carmeliet et al. 1999; Ruhrberg et al. 2002; Stalmans et al. 2002; Gerhardt et al. 2003). This enabled us to analyze interneuron migration in an in vivo context in which the vascular scaffolding was perturbed. Surprisingly, we observed a modest, but significantly increased number of interneurons in the *Vegfa*^120/120^ cortex at E14.5 despite the aberrant vascular network (Figs. 3A,B & 4B), which was neither attributed to altered MGE progenitor proliferation or changes in their viability (Supplementary Fig. 4). Importantly, this indicated that the tangential migration of interneurons was not impeded by an altered vascular network at this stage and suggested that interneurons may respond to chemotropic sources of Vegfa. Support for this was provided by our expression studies showing that interneurons express cognate VegfaR1 and Nrp1 receptors (Fig. 1*C-G*) and by our in vitro migration assays which showed that early and late-migrating interneurons differentially responded to Vegfa isoforms (Fig. 5*A*). Pertinently, we found that Vegfa120 promoted the migration of early, but not later-migrating interneurons, consistent with our in vivo observations of an increased and decreased number of interneurons which reached the dorsal cortex at mid (E14.5) and late (E18.5) stages (Fig. 3). Further, we showed for the first time that Vegfa splicing occurred in a temporal and region-dependent manner in blood vessels, suggesting that they provide spatially segregated cues in the embryonic forebrain (Fig. 5*B,C*). We next tested whether interneurons responded to a chemotropic source of vascular-Vegfa by selectively depleting Vegfa from Tie2^+^ blood vessels. We found a significant reduction in cortical interneurons at mid stages, a decrease in their spatial proximities with the vasculature and their defective positioning in the CP at late stages, despite no significant changes in the vasculature (Figs. 6 and 7). These changes occurred in the absence of alterations in the proliferation or survival of interneurons (Raab et al. 2004; Cariboni et al. 2011). Moreover, endothelial depletion of Vegfa did not affect the generation of IPs in the dorsal cortex at early-mid stages or in their expression of Sdf1 (Fig. 7), showing that the reduction in cortical interneurons is not due to indirect effects of vascular-Vegfa on IPs at this time. Together this suggests that vascular-Vegfa specifically influences interneuron migration at early-mid phases of cortical development, findings which were corroborated by our in vitro assays in which Vegfa isoforms and pial-derived Vegfa directly stimulated interneuron migration (Fig. 5*A* and Fig. 8*E*). It was further substantiated by our models in which addition of chemoattractants to blood vessels more closely recapitulated the proximity of interneurons to the vasculature in vivo (Fig. 8 and Supplementary Fig. 5). While the intracellular mechanisms by which Vegfa promotes the migration of VegfaR2^+^ cerebellar (Ruiz de Almodovar et al. 2010) and Nrp1^+^ facial branchiomotor neurons (Tillo et al. 2015) are not known, Vegfa/VegfaR2 signaling drives actin-based VEC motility (Rousseau et al., 2000) to modulate their focal cell adhesion assembly and turnover (Abedi and Zachary 1997; Eliceiri et al. 2002). Future studies in which VegfaR1 is impaired in migrating interneurons are required to directly address how Vegfa signaling impacts interneuron migration, and to assess if this may similarly modulate their motility and proximity with blood vessels by regulating their adhesive interactions with the extracellular matrix and the vascular basement membrane. Nevertheless, our data shows that vascular-Vegfa promotes interneuron migration and their positioning in the developing forebrain at early stages of their migration in vivo*.*

Our findings that endothelial ablation of Vegfa at late stages did not recapitulate the interneuron deficit observed in the *Vegfa*^*120/120*^ mutants showed that the tangential migration of interneurons at late phases was not dependent on vascular-Vegfa. Indeed, consistent with previous observations (Haigh et al. 2003), we found that Vegfa was also expressed by pallial progenitors and pyramidal neurons (Fig. 1*A*), and that there were defects in neurogenesis in the dorsal cortex at this time (Fig. 9). While IPs have been shown to promote interneurons’ tangential migration to the cortex through their secretion of *Sdf1* (Tiveron et al. 2006; Sessa et al. 2010; Abe et al. 2015), we surprisingly found no gross alterations in the expression of this chemokine in the E17.5 *Vegfa*^*120/120*^ mutant cortex, despite the reduction in Tbr2^+^ IPs at this time. Further studies are required to quantitatively assess potential changes in Sdf1 levels in these mutants; however, our current analysis suggests that either a small reduction in Sdf1 is sufficient to impede the tangential migration of later-born interneurons into the cortex or that other as-of-yet unidentified factors secreted by IPs could be involved. While the constitutive expression of the diffusible *Vegfa*120 chemoattractant from MGE progenitors could potentially inhibit interneuron dispersion towards the dorsal cortex and explain their increased subpallial accumulation in the *Vegfa*^120/120^ mutants, our findings that late-migrating interneurons were not responsive to the *Vegfa*120 isoform in vitro (Fig. 5*A*) argue against this. Future work selectively depleting *Vegfa* from MGE progenitors, pallial intermediate progenitors and from postmitotic pyramidal neurons are required to address a potential role for neural-*Vegfa* in coordinating interneuron migration at late stages of corticogenesis. Our data, nevertheless, support a role for vascular-secreted factors and Vegfa isoforms in influencing the correct tangential migration and intracortical positioning of interneurons at early-mid stages of corticogenesis in vivo*.* Understanding the functional consequence of interneuron deficits in Vegfa120 adult mice would be important, however this remains difficult as only 0.5% of mice survive to postnatal day 12 due to cardiac defects (Carmeliet et al. 1999). Future studies however could address whether the defective migration of VegfaR1^+^Nrp1^+^ interneurons in *Tie2Cre;Vegfa*^*fl/fl*^ mutant cortex impacts their assembly into neural circuits in the adult cortex, and elucidate the possible functional and behavioral significance of this population of interneurons.

This is important given that *Vegfa* is expressed in vECs and neural cells in the fetal human brain (Virgintino et al. 2003), and because *Vegfa* polymorphisms and its downregulation have been implicated in schizophrenia and other mood disorders (Fulzele and Pillai 2009; Gao et al. 2015). While it is not known if human cortical interneurons express *Vegfa* receptors, these disorders are associated with interneuron deficits (Inan et al. 2016). It remains to be established whether aberrant vascular-*Vegfa* signaling underlies perturbed interneuron migration and positioning during human fetal development which, in turn, may result in defective cortical neuronal circuitry reported in these neuropsychiatric disorders. In addition, the maturation of human fetal forebrain blood vessels is dependent on *Vegfa*, and has been shown to be compromised in prematurely born infants, which increases their susceptibility of developing periventricular leukomalacia (Licht et al. 2015). This devastating disorder results in severe mental retardation and in the extensive necrosis within the subpallium which impairs the generation of early-born populations of GABAergic interneurons. Our data raise the possibility that aberrant *Vegfa*-isoform expression in the developing forebrain could additionally perturb the migration and positioning of later-migrating interneurons through neural-derived or indirect effects of *Vegfa*. Whether defective *Vegfa* signaling alters cortical interneuron numbers and positioning during human fetal development remains to be seen. However, the present work points to a potential novel mechanism by which alterations in vascular-secreted guidance cues and in *Vegfa* signaling could underlie the pleiotropic origins of human neurodevelopmental disorders.

## Supplementary Material

Supplementary Data
