# Supplementary material for: Vascular-Derived Vegfa Promotes Cortical Interneuron Migration and Proximity to the Vasculature in the Developing Forebrain
Source: Cereb Cortex. 2018 Apr 18;28(7):2577–93. doi: 10.1093/cercor/bhy082 (PMC5998991; doi:10.1093/cercor/bhy082)
Supplement: Supplementary Data [file bhy082suppl_1.zip › CerebralCortex_SupplementaryMethods_FigCaptions.docx]

**Supplementary Materials & Methods**

***Dissociated MGE cultures***

Dissociated cell cultures were prepared from either embryonic mice or Sprague Dawley albino rat brains as described previously (Cavanagh et al., 1997). Briefly, the MGE was dissected out in cold artificial cerebral spinal fluid (ACSF) under a stereomicroscope, trypsinised in 0.05% trypsin (Merck) with 100 μg/ml DNaseI (Roche) in Neurobasal medium (ThermoFisher Scientific) for 15 min at 37 ºC. Trypsinisation was quenched with neutralization medium containing 10% of FBS (ThermoFisher Scientific) in Neurobasal medium for 5 min at 37 ºC, and subsequently mechanically dissociated. Homogenous cell suspensions were subsequently pelleted by centrifugation at 1,000 x g for 3 min and cells resuspended in low-serum Optimem media (ThermoFisher Scientific) containing B27 supplement (ThermoFisher Scientific) and 100 µg/ml penicillin/streptomycin for Boyden assays.

***Matrigel explants***

Matrigel explants were performed as previously described (Hernandez-Miranda et al., 2011b). COS7 cells or mechanically dissociated pial cells were embedded in 15 μl droplets of 2% agarose in DMEM (2 x 10^7^/ml) onto 13 mm coverslips coated with 10 μg/ml poly-L-lysine and 10 μg/ml laminin in 24-well plates. These were co-cultured with 4 small pieces of E15.5 rat MGE, all embedded in Matrigel solution (BD Biosciences) and cultured in DMEM/F12 media with Glutamax (ThermoFisher Scientific) supplemented with B27 supplement (ThermoFisher Scientific) and 100 µg/ml penicillin/streptomycin. Explants were cultured for 2 d *in vitro* in a sterile incubator (37°C, 5% CO_2_). Cell migration from MGE explants was assessed by measuring the distance migrated from proximal and distal side of the explants. In some experiments, VEGF receptor inhibitor Axitinib (1.2 nM, Tocris bioscience) was added to the wells. All explants were repeated in four independent experiments.

***Quantification of distribution of interneurons, microglia, CC3 and PH3 cell counts***

Calbindin, Reelin, Lhx6, and Gad67 positive cells were counted in 400 μm coronal strips spanning the thickness of the dorsal-lateral cortex at rostral and middle (along the rostro-caudal axis) levels of the forebrain at E14.5 and E18.5 in *Vegfa^120/120^, Vegfa^165/165^* and respective *Vegfa^+/+^* controls (minimum of 8 sections from n=3 brains for each condition)*,* and at E13.5 (n=2 brains for each), E14.5 (n=2 brains for each) and E18.5 *Tie2Cre;Vegfa^fl/fl^* and respective *Vegfa^fl/fl^* controls (n=3 for each genotype, minimum of 3 sections from each brain for each condition). Counts for E13.5 were combined with E14.5 for statistical analysis of *Tie2Cre;Vegfa^fl/fl^* and respective *Vegfa^fl/fl^* controls. In all counts, the experimenter did not know the condition of the animal. Strips were divided into 6 bins arranged parallel to the pial surface that corresponded to the different layers of the developing cortex delineated by Dapi staining for immunostainings (MZ, CP, SP, IZ, SVZ/IZ, VZ), from bin1 (VZ) to bin 6 (MZ).

Proliferation analysis in *Vegfa^120/120^* and *Vegfa^+/+^* forebrains was carried out by quantifying apical progenitors lining the VZ, presented as PH3 labelled cells per 100 μm. Basal progenitors in the SVZ were presented as PH3 labelled cells per 9x10^4^ μm^2^. Basal progenitors here were defined as any cell more than three cells width away from ventricle surface. In *Tie2Cre;Vegfa^fl/fl^* and *Vegfa^fl/fl^* animals, the germinal MGE VZ and SVZ regions were delineated by Bizbenzamide nuclear staining and apical VZ progenitors defined as PH3^+^ counts normalised per 100 μm. PH3+ counts in the MGE SVZ are normalised by the domains surface area, measured by outlining each domain with the polygonal tool in Fiji and pixels calibrated to µm. Counts at E13.5 and E14.5 were grouped together for *Tie2Cre;Vegfa^fl/fl^* and respective *Vegfa^+/+^* controls by normalising mutant counts relative to controls which were set at 100%. PH3^+^ which were adjacent and in contact to IB4^+^ filopodia extending to the MGE VZ, or in contact with IB4^+^ blood vessels in the MGE SVZ were counted and expressed as a percentage of total PH3^+^ cells in each progenitor domain. CC3 counts were made in sections taken through the brains of *Vegfa^+/+^* and *Vegfa^120/120^* embryos at E14.5 and E18.5 also stained for Iba1, Calbindin, and IB4. The number of CC3 labelled cells was counted in 300 µm bins in the cortex as described above. Counts made in the VZ of both the LGE and MGE are expressed as PH3^+^ cells/ 100 µm and in the LGE and MGE SVZ normalised per surface area (minimum of 8 sections from each of 3 animals for each condition). CC3^+^ cells colocalising with IB4^+^ blood vessels or Calbindin^+^ cells were scored, as well as Iba1^+^ cells in direct contact with double labelled IB4^+^CC3^+^ blood vessels.

***Proliferation, apoptosis and survival assays***

Dissociated rat E15.5 MGE cultures were incubated overnight in the presence or absence of Vegfa120 (10 ng/ml) (R&D Systems). The following day, 10 mM BrdU was added to the medium for 2 h after which, cells were washed, fixed with PFA, immunostained for BrdU and nestin or Pax6 to identify different progenitor types. The percentage of cells immunoreactive for BrdU and nestin or Pax6 was counted using a *×40* objective lens in nine fields of view for each coverslip. At least three coverslips were evaluated from each animal for each time point and treatment, and significance was established using one-way ANOVA. To determine the effect of Vegfa on survival, E15.5 cultures were prepared as above, without N2 or B27, which are required for the normal maintenance of cortical and MGE cells, again in the presence or absence of Vegfa120 (10 ng/ml). The next day, cells were washed and fixed with PFA, immunostained for CC3 and counterstained with DAPI. Cell counts were made with a *×40* objective in nine fields of view for each sample carried out in quadruplicate.

***Analysis of vasculature***

Sections taken through the brains of *Vegfa^120/120^, Vegfa^165/165^ Vegfa^188/188^* and respective *Vegfa^+/+^* controls*;* and in *Tie2Cre;Vegfa^fl/f^* and *Vegfa^fl/fl^* embryos at E14.5 were immunostained for IB4. Confocal tile-scan images acquired with the x40 objective, comprising 21 µm z-stack projections were binarised according to the same intensity threshold, and masks created and the total area occupancy of signal measured, together with IB4^+^ particle size to estimate the surface area and size or blood vessels using Fiji’s (NIH) Particle Analysis plugin. Automated and manual measurements were calculated in 300 µm bins or areas of 9x104 μm^2^ of the cortex and MGE (minimum of 8 sections from each of 3 animals for each condition except for *Vegfa^188/188^* in which only one animal was available for each), respectively. Additional vascular surface was summed in all optical slices of the z-stack using the abovementioned bespoke plugin.

***Quantification of interneuron and blood vessel association***

Sections taken through rostral and middle levels of the brains of *Gad67-Gfp^+^* embryos at E13.5, E15.5 and E17.5 (minimum of 2 sections from 3 brains for each age); *Tie2Cre;Vegfa^fl/fl^* and *Vegfa^fl/fl^* at E13.5 (minimum of 6 sections from n=2 brains for control and n=3 mutant); of *Vegfa^120/120^, Vegfa^165/165^, Vegfa^+/+^* (minimum of 3 sections from n=3 brains for control and mutant)*, Tie2Cre;Vegfa^fl/fl^* and *Vegfa^fl/fl^* embryos at E14.5 (minimum of 3 sections from n=3 brains for each condition) were immunostained for IsolectinB4 and Calbindin or, for *Gad67-Gfp^+^* brains, with Gfp. Confocal tiled-scan images were acquired with the x40 oil objective from the dorsal to lateral extent of the cortex at rostral and middle levels of the forebrain, with a total z-stack of 20 µm and a 1 µm z-step interval, using the Diode, Argon and Helium lasers at 405, 488, 568 and 633 nm. The minimum distance of the centroid of Gad67^+^ interneurons to the closest IB4-labelled vascular surface was calculated using a bespoke ImageJ plugin (designed by D.Ciantar, Imaging Facility UCL, London) with images threshold according to signal intensity and predicted cell-size. All intra-cellular distances were verified by opening merged masks with Imaris 8 (Bitplane) and erroneous traces removed from the data set.

**Supplementary Figure Captions**

**Supplementary Figure 1. Migrating cortical interneurons are located close to sources of Vegfa in the dorsal cortex at E17.5 and express cognate Vegfa receptors.** (*A*) *In-situ* localisation of *Vegfa* transcripts in the VZ, forming cortical plate and meninges of the dorsal cortex of the E17.5 Gad67-Gfp+ mouse forebrain, adjacent to migrating Gad67-Gfp+ interneurons. (*B*) Expression of Vegfa protein, the vascular endothelial marker IB4, and the pericyte-specific Pdgfrß/Cd140b protein in the E17.5 dorsal-lateral cortex with its co-localisation in blood vessels shown in inserts. (*C*) Expression of VegfaR1 in migrating Gad67-Gfp^+^ interneurons (white arrows) in the lower LIZ/SVZ, and subplate of the dorsal cortex at E17.5.

**Supplementary Figure 2. Cortical interneurons do not express VegfaR2 and VegfaR3.** (*A,B*) Immuno localisation of VegfaR2 (A) and VegfaR3 (B) receptors in the E13.5 Gad67-Gfp+ mouse forebrain, with adjacent panels showing high magnification images of the dorsal cortex.

**Supplementary Figure 3. Altered number and distribution of cortical interneurons in Vegfa120 ubiquitously expressing transgenic knock-in mouse cortex** (*A*) Histograms show mean total number of Calbindin+ interneurons in the dorsal cortex of *Vegfa^120/120^* and *Vegfa^+/+^* mouse forebrains (Graphs show mean values ± SEM; n=3 for each) with line-graphs showing the normalised intra-cortical distributions of *Gad67^+^*, Calbindin+ interneurons (n=3 for each)(T-test, *p≤0.05, **p≤0.01, p≤0.001) (*B*) In-situ hybridization for interneuron marker *Gad67* in the cortex of E18.5 *Vegfa^120/120^* and *Vegfa^+/+^* mouse forebrains.

**Supplementary Figure 4. MGE progenitor proliferation and cortical interneuron survival are unaltered in the *Vegfa^120/120^*** **mutant forebrain.** (*A*) Graphs show number of proliferating PH3^+^ progenitor cells normalised per 100µm length of the ventricular (VZ) and per 90,000 µm^2^ surface area of the subventricular (SVZ) germinal domains of the E14.5 MGE and dorsal cortex of *Vegfa^+/+^* and *Vegfa^120/120^* knock-in mouse forebrains (n=3 *Vegfa^+/+^;* n=4 *Vegfa^120/120^*). (*B*) Immunolabelling of dissociated E15 rat MGE cells (equivalent to E13.5 in the mouse) for the neural progenitor marker, Nestin, and the bromodeoxyuridine (BrdU) proliferative marker to label cells entering the DNA synthesising S-phase of the cell-cycle over a 2hr period, in cultures treated with control media and Vegfa164 recombinant proteins for *1div.* Graphs show percentage of proliferating Nestin^+^BrdU^+^ cells out of total Nestin^+^ MGE progenitors. (*C*) Images of the MGE of E14.5 *Vegfa^+/+^* and *Vegfa^120/120^* knock-in mouse forebrains immunolabelled for the apoptotic caspase-3 (CC3^+^) markers, and blood vessels labelled with IB4^+^ with arrows and high magnification panels showing localisation of CC3^+^ cells in IB4^+^ blood vessels. (*D*) E15 rat MGE cells cultured for 24 h in the absence of pro-survival B27 and N2 factors (left panel), or with the addition of Vegfa164 (right panel) human recombinant proteins, and immunolabelled for CC3^+^ proteins and counterstained with the nuclear marker Dapi. Graphs show mean % of total Dapi+ cells which colabelled with the apoptotic CC3^+^ marker for each condition. (n=3 experiments) (Graphs show mean values ±SEM).

**Supplementary Figure 5.** (*A*) Top graph shows mean number and distribution of Calbindin^+^ interneurons in the dorsal cortex of E13.5/14.5 combined *Vegfa^fl/fl^* control relative to *Tie2Cre;Vegfa^fl/fl^* mutant forebrains. Bottom graphs show the sum vascular perimeter lengths, mean vascular size and vascular surface area occupancy in the cortex of E13.5 *Vegfa^fl/fl^* control (n=2) and *Tie2Cre;Vegfa^fl/fl^* mutant animals (n=2), calculated with the bespoke plugin. (*B*) Bar charts show the number of Calbindin+ interneurons located at mean minimum distances (µm) from the closest IB4+ vascular surface in the dorsal cortex of E14.5 *Vegfa^fl/fl^* control relative to *Tie2Cre;Vegfa^fl/fl^* mutant forebrains, measured using the bespoke plugin. (*C*) Schematics show how the *in-vivo* representations of blood vessels and interneurons (left panels) were modelled by pairs of lines and spots, respectively (right panels). Both objects were randomly distributed in a 3-d volume space and the mean minimum intra-object distances calculated in the same manner as when analysing the *in-vivo* interneuron-vascular distributions. Graphs show how changing (*C*) blood vessel (BV) surface area (SA) or (*D*) vascular area fraction occupancy influences the relative distributions of interneurons with vascular surfaces. (Graphs show mean values ±SEM). (PPL/MZ, preplate/marginal zone; CP, cortical plate; SP, subplate; IZ, intermediate zone; VZ, ventricular zone).

**Supplementary Figure 6. Cortical interneuron numbers are unchanged in the cortex of *Vegfa^165/165^* E14.5 mutant forebrain with normal vascular networks.** (*A*) IB4^+^ labelling of vasculature and immunolabelling of Calbindin^+^ interneurons in coronal sections from E14.5 *Vegfa^165/165^* (n=3) knock-in mouse forebrains. Bar graphs show automated analysis of IB4^+^ vascular surface area and IB4^+^ pixel size calculated from images binarised according to a set thresholded IB4^+^ signal intensity. Line graphs show cumulative distribution of all mean minimum distances calculated between Calbindin^+^ cell-centroid and the closest IB4+ vascular surface using the bespoke plugin (see Methods). (*Vegfa^165/165^* n= 2273 cells & *Vegfa^+/+^;* n=1238 cells; KS-test p≤0.0001). *(B)* In-situ hybridization for interneuron-specific *Lhx6^+^ mRNA* in the cortex of E14.5 *Vegfa^165/165^* knock-in mouse forebrain. Bar charts shows mean total numbers of *Lhx6+* interneurons in the dorsal cortex of Vegfa165 isoform-specific mutant mice (*Vegfa^165/165^*, n=3 for each)(T-test, **p≤0.01). (Graphs show mean values ±SEM).
