# Supplementary figures and images for: Vascular-Derived Vegfa Promotes Cortical Interneuron Migration and Proximity to the Vasculature in the Developing Forebrain
Source: Cereb Cortex. 2018 Apr 18;28(7):2577–93. doi: 10.1093/cercor/bhy082 (PMC5998991; doi:10.1093/cercor/bhy082)

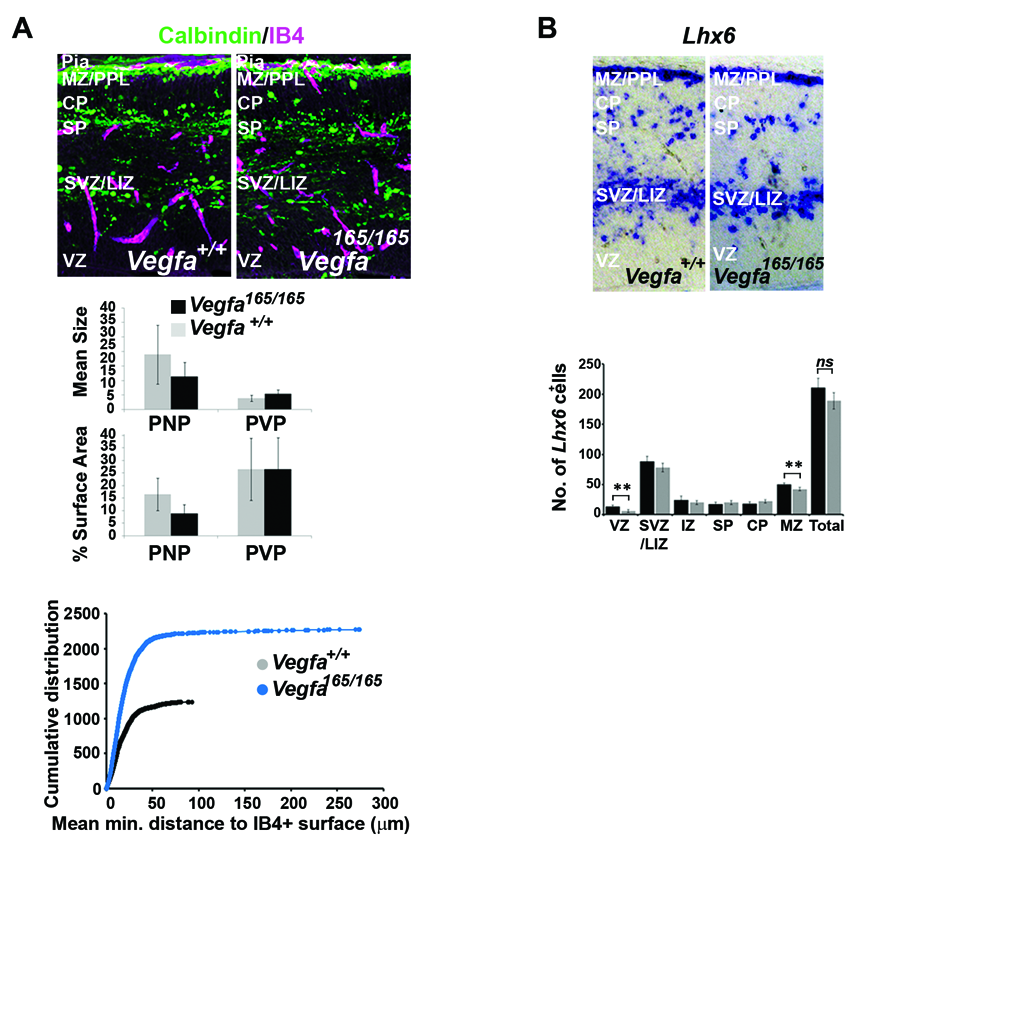

Supplement: Supplementary Data [file bhy082suppl_1.zip › Suppl_Fig_6-flat.tif]

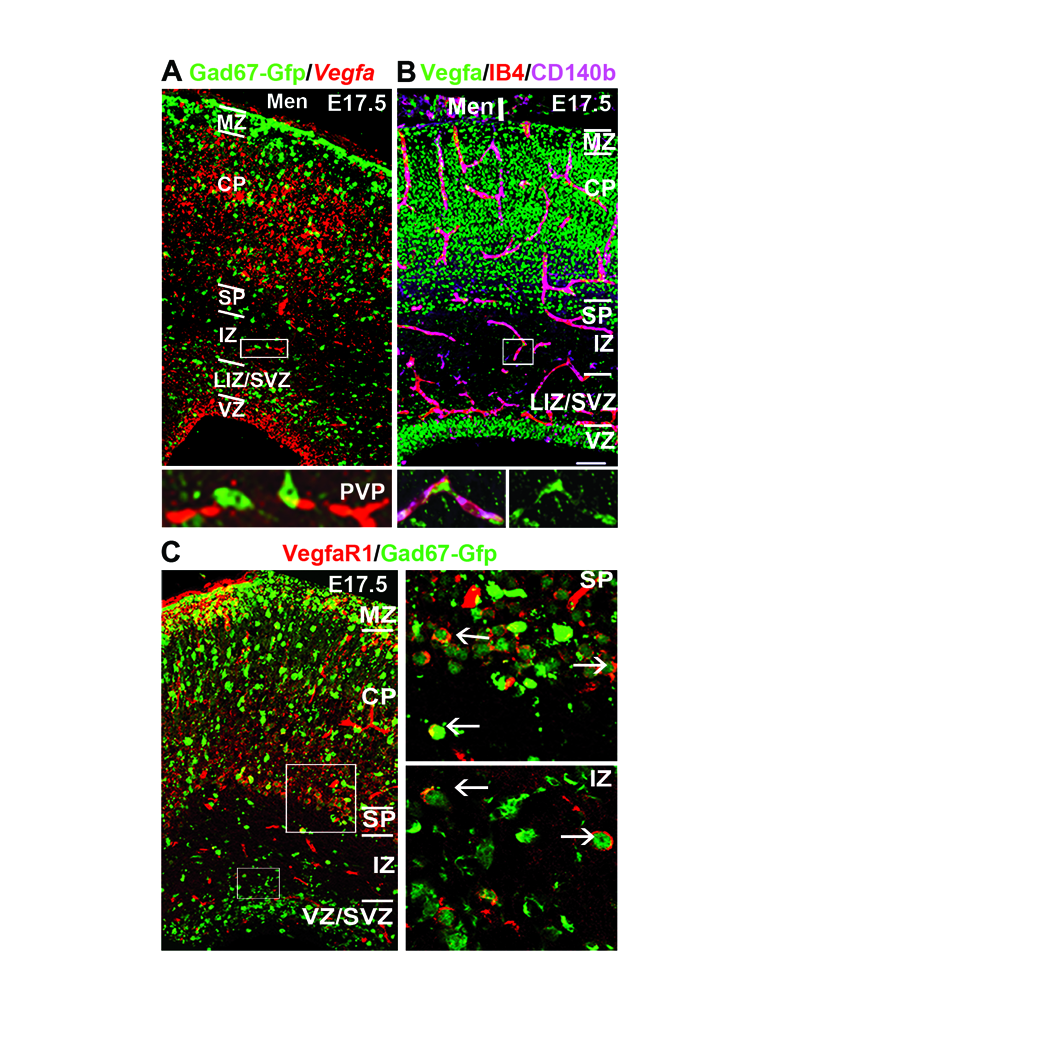

Supplement: Supplementary Data [file bhy082suppl_1.zip › Suppl_Fig_1-flat.tif]

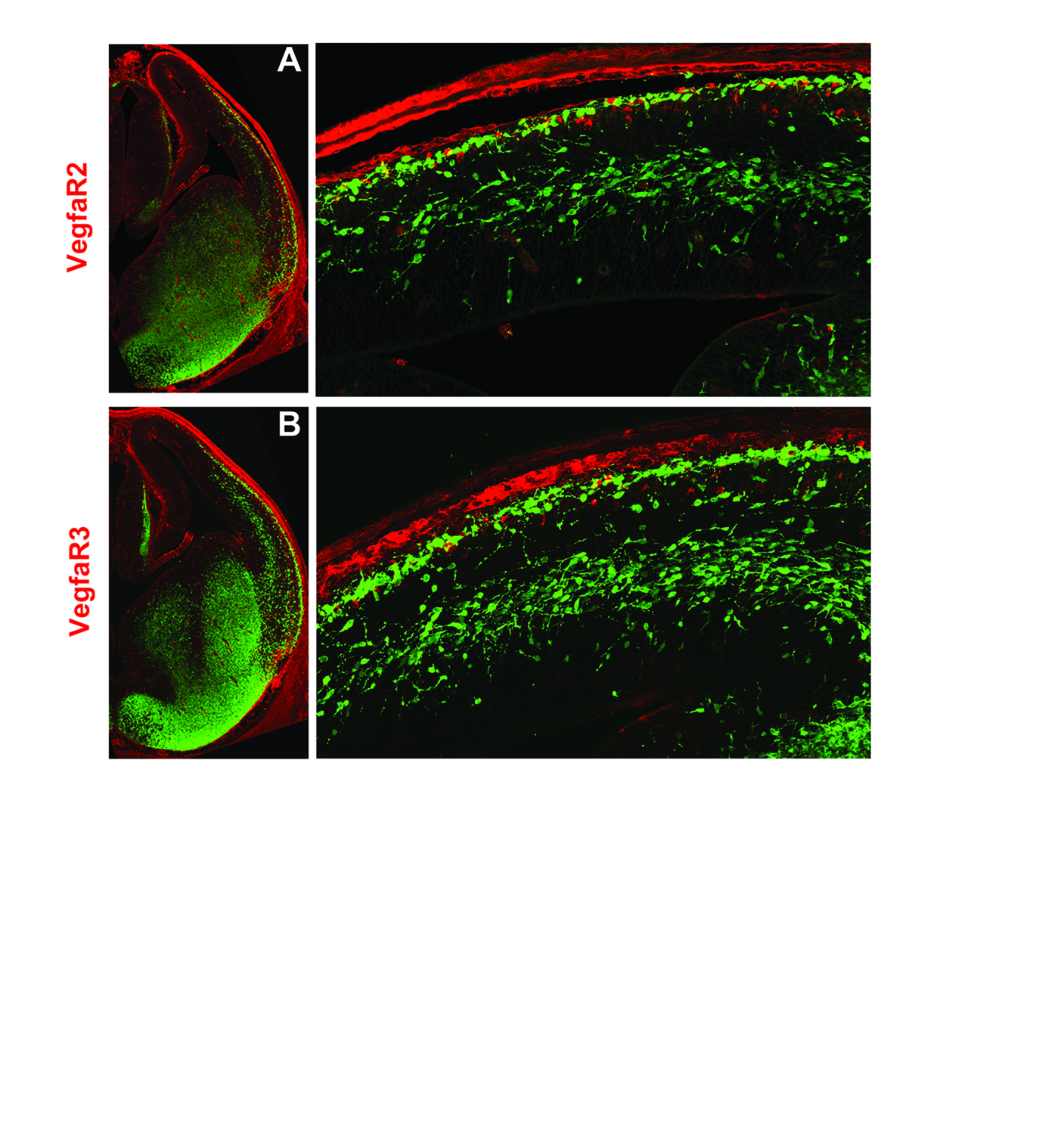

Supplement: Supplementary Data [file bhy082suppl_1.zip › Suppl_Fig_2-flat.tif]

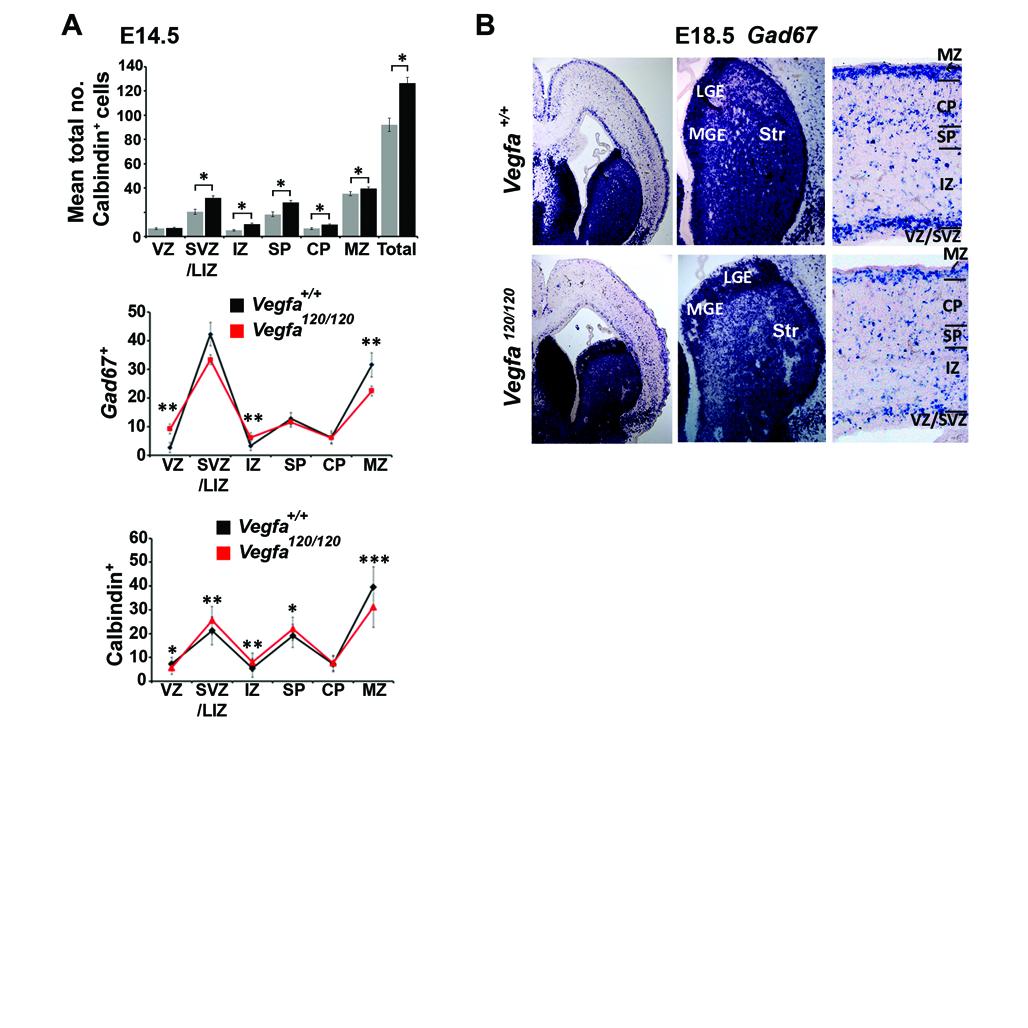

Supplement: Supplementary Data [file bhy082suppl_1.zip › Suppl_Fig_3.tif]

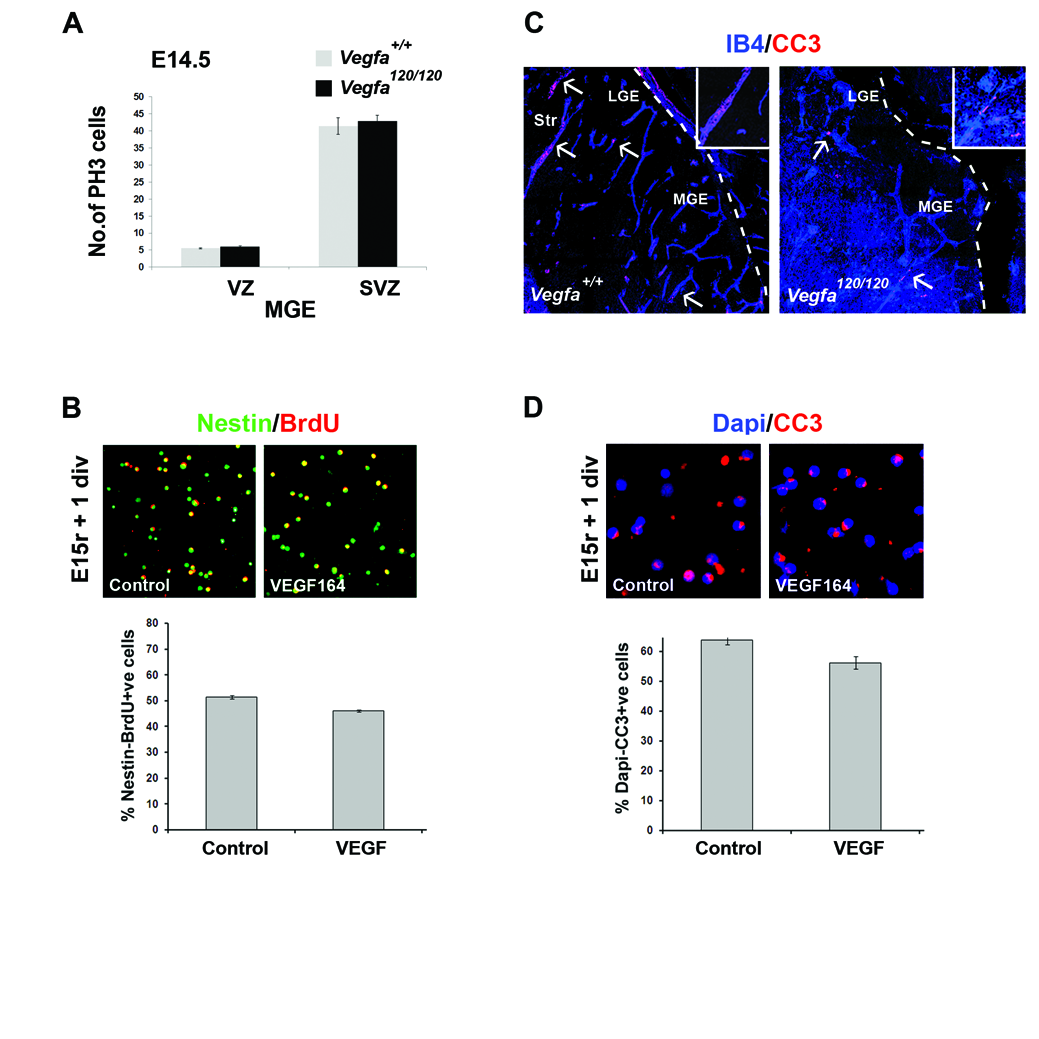

Supplement: Supplementary Data [file bhy082suppl_1.zip › Suppl_Fig_4.tif]

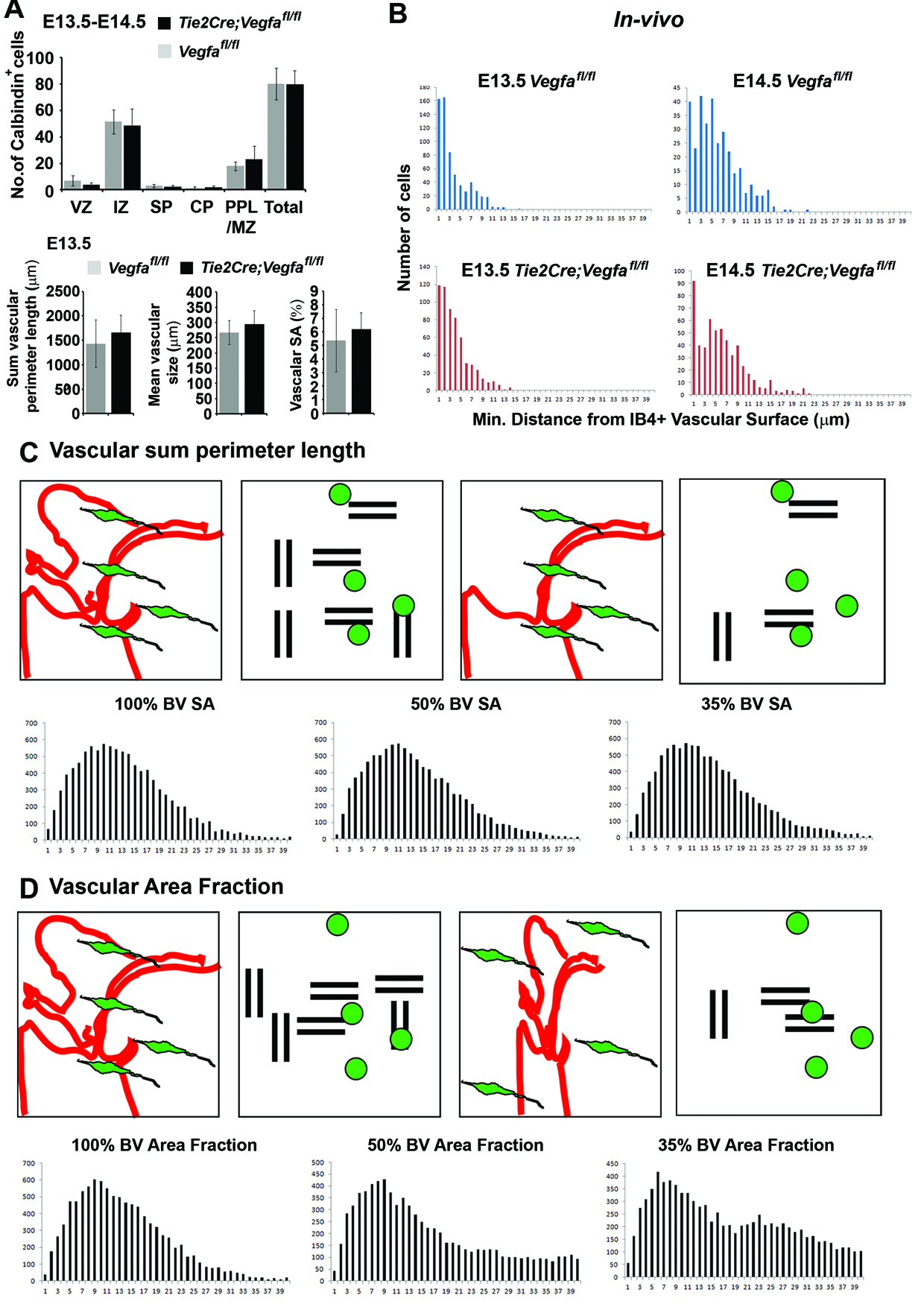

Supplement: Supplementary Data [file bhy082suppl_1.zip › Suppl_Fig_5-flat.tif]
